# Shifting towards optimized healthy and sustainable Dutch diets: impact on protein quality

**DOI:** 10.1007/s00394-023-03135-7

**Published:** 2023-03-23

**Authors:** Samantha N. Heerschop, Argyris Kanellopoulos, Sander Biesbroek, Pieter van ‘t Veer

**Affiliations:** 1 Division of Human Nutrition and Health, Wageningen University and Research, Postbox 17, 6700 AA Wageningen, Gelderland The Netherlands; 2 Operations Research and Logistics Group, Wageningen University and Research, Wageningen, Gelderland The Netherlands

**Keywords:** Protein transition, Protein quality, Diet optimization, Sustainable diets, Dutch diets, Meal-based approach

## Abstract

**Purpose:**

To reduce the environmental impact of Western diets, a reduction of meat consumption and a substitution by plant-based protein sources is needed. This protein transition will affect the quantity and quality of dietary protein. Therefore, the aim of this study was to evaluate the protein adequacy of diets optimized for nutritional health and diet-related greenhouse gas emission (GHGE).

**Methods:**

Data from 2150 adult participants of the Dutch National Food Consumption Survey were used, with diet assessed using two non-consecutive 24 h dietary recalls. Utilizable protein of current diets per day was based on meal composition and the Protein Digestibility-Corrected Amino Acid Score and was compared to protein requirements. Optimized diets were derived as linear combinations of current diets that minimized GHGE and maximized the Dutch Healthy Diet 2015 score, with/without constraints to keep dietary change within 33% of current consumption. Protein adequacy was evaluated in both current and optimized diets.

**Results:**

In all age and gender strata, the healthiest diets had higher GHGE, the most sustainable diets had the lowest dietary quality, though higher than current diets, and protein adequacy remained sufficient. When limiting dietary change to 33% of current consumption, in the most promising trade-off diet GHGE was reduced by 12–16%. The current diet provided 1.4–2.2 times the required amount of utilizable protein.

**Conclusion:**

These results suggest that a realistic aim for the next decade might be to reduce diet-related GHGE to 12–16% of the current levels without compromising protein adequacy and diet quality. To achieve global targets, upstream food system transformations are needed with subsequent dietary changes.

**Supplementary Information:**

The online version contains supplementary material available at 10.1007/s00394-023-03135-7.

## Introduction

The current Western diet is associated with an increased incidence of non-communicable diseases such as coronary heart disease, diabetes and cancer [1]. Next to the impact on human health, diet also influences the environment, as global food production and consumption are responsible for about a quarter of total greenhouse gas emissions (GHGE) [2]. Hence, there is an urgent need for healthier and more environmentally sustainable diets.

Several proposed sustainable diets show an increased ratio of plant- versus animal-based products [3, 4]. As plant products are known for their lower protein quality, the shift towards more plant-based diets asks for a careful evaluation of protein adequacy in these diets [5–7]. Until now, many studies have evaluated only the protein quantity of optimized healthy and sustainable diets [8–13]. They all found that the total protein was lower in the modelled diets, but still adequate. However, when shifting to a more plant-based diet, not just protein quantity, but also protein quality becomes important.

Protein adequacy depends on the net protein utilization (NPU) within meals, i.e. a combination of digestibility and the biological value (BV) of the protein [14, 15]. Digestibility includes the percentage of ingested nitrogen that is absorbed by the body, while the BV is the percentage of absorbed nitrogen that can be utilized by the body. The BV depends largely on the limiting indispensable amino acid (IAA) in the digested protein, i.e. the one that is shortest in supply relative to body requirements for absorbed amino acids and the ratio between IAAs and dispensable amino acids (DAAs) [16]. The NPU of an average diet is currently estimated to be 47% and protein requirements are based on this NPU [14, 17]. However, the NPU may differ between diets that vary in ratios of animal- and plant-based foods [6, 7, 18]. As the more sustainable, and thereby more plant-based, diets may have lower NPUs, it is important to evaluate modelled sustainable diets on their protein quality.

To provide healthy and sustainable dietary advice, food-based diet models have been used to optimize nutritional health and/or environmental sustainability using linear combinations of food items [3, 4, 19]. In these models acceptability constraints are used to restrict the food item intakes to such observed in current meals and diets to calculate diets that account for cultural preferences and dietary habits of consumers. Defining acceptability constraints is a challenging process that often involves expert knowledge and a substantial degree of subjectivity. To overcome this, Kanellopoulos et al. [20] developed a benchmarking model that used linear combinations of whole diets of peers (rather than combinations of foods) [20, 21]. By benchmarking current diets, the model provides for each diet in the population a set of alternative diets (expressed as linear combinations of current diets) that are healthier than the current diet. In this research, we calculate for each individual the healthiest possible diet, which is still a combination of diets of other individuals in the population. To propose sustainable, healthy, acceptable, realistic and preferable (SHARP) diets, this study extended the model by explicitly incorporating a healthy diet indicator to enable optimization of the health score of the calculated diet. For a health indicator, it is important that the holistic health properties of the diet are represented, rather than nutrients only [22]. Food products contain many nutrients and non-essential bio-actives that are healthy and prevent non-communicable diseases, but cannot be measured reliably. For this reason, we used the Dutch Healthy Diet index 2015 (DHD15) which is based on food products and developed to meet both nutrient requirements and prevent non-communicable diseases [23].

To get an insight into the protein quality and adequacy of healthy and sustainable diets that are acceptable to consumers, the present study optimized diets using the Dutch National Food Consumption Survey (DNFCS) 2012–2016 on nutritional health and environmental impact and evaluated its protein adequacy.

## Methods

### Study population

The study population used for the optimization consisted of participants of the DNFCS 2012–2016 [24]. The total survey included 4313 persons (response rate 65%) and was conducted between 2012 and 2016. The present study only included adults: 2150 men and women aged 18–79 years. The nationally representative study population was drawn from a consumer panel of the market research agency KANTAR TNS. An age–gender random sampling strategy was applied. Furthermore, the representativeness of region, address density and education was taken into account.The DNFCS was conducted according to the guidelines of the Helsinki Declaration. Because of the non-invasive measurements in this survey, the Medical Ethical Committee of the University Medical Centre Utrecht, the Netherlands, concluded that the study did not need to be evaluated according to the ‘Medical research on human act’ (WMO) (reference number 12-359/C). Therefore, written informed consent was not required for this survey at the time of data collection. A detailed description of the recruitment of the study population is described elsewhere [24].

### Dietary assessment

The food consumption data consisted of two non-consecutive 24 h dietary recalls for people aged 1–70 years. Standardized interviews were conducted by trained dieticians using the GloboDiet (former EPIC-soft©) computer program, developed by the International Agency for Research on Cancer, Lyon, France [25]. GloboDiet comprises among others information on food consumption occasions with time and place of consumption. We used this information to define meals. To obtain consumption information independent of possible fluctuation in dietary patterns per season, the 24 h dietary recalls were spread over seasons and days of the week, both week and weekend days.

### Food composition

Food composition of the consumed products was derived from the Dutch Food Composition Database to calculate the intake of energy and nutrients (NEVO-online version 2016/5.0) [26]. Amino acid data were not yet available in the NEVO database. Therefore, we extended the NEVO food composition database with amino acid values for products that were consumed in the DNFCS 2012–2016, based on the Danish (Frida), American (USDA), English (Mccance and Widdowson) and the Japanese food composition tables. Besides, we defined a protein digestibility (PD) factor per food group using literature [15, 27–35]. The full procedure and the references to all food composition tables used are described in Online Resource 1. Food items from the DNFCS 2012–2016 were grouped into 28 food groups adapted from the GloboDiet food group classification. Online Resource 2 shows detailed information about the products included in each of these food groups. In this paper, food items from the DNFCS 2012–2016 are presented in eight food groups, also depicted in Online Resource 2. For the present study, food intake is standardized to the amounts consumed per 2000 kcal, which preserves the relative composition of the diet and allows to compare this between population subgroups for e.g. age and gender.

### Protein quality and adequacy

Protein adequacy involves three components: (1) meeting the requirements for the IAAs according to the reference profile, (2) meeting the requirements for the DAAs, i.e. the appropriate ratio of IAAs and DAAs and (3) meeting the daily N-requirement. The first two components relate to protein quality and are derived from protein digestibility and amino acid (AA) reference profiles [16]. To evaluate the adequacy of protein intake, the third component is derived from nitrogen balance studies and includes the total amount of AA (or protein) consumed. As detailed below, this procedure follows the recommendation by the World Health Organization to evaluate the intake of AA and total protein as separate nutrient requirements, where the lowest component is the limiting factor.

The first component involves the IAA profile of the diet. This was calculated for each meal using the Protein Digestibility-Corrected Amino Acid Score (PDCAAS) and represents the proportion of each IAA in the reference profile that can be digested (PD) and subsequently utilized (AAS) for protein synthesis in the body. The reference pattern for the adult population was used. The formula of calculating PDCAAS for a meal is as follows [16]:

1 $${\text{PDCAAS}}_{{{\text{meal}}}} { } = {\text{digestibility }}\left( {{\text{wPD}}} \right){\text{*MIN}}\left( { \frac{{\text{mg of IAAi in 1g test protein}}}{{\text{mg of IAAi in the reference pattern}}} , 1} \right)\left( {{\text{AAS}}} \right),$$

where wPD is the weighted protein digestibility of the meal, with weights for the amount of protein in each food product in the meal. The part between brackets is usually called the amino acid score (AAS) and represents the ‘biological value’ (BV). In the AAS, the ‘test protein’ is the protein as provided by meals in the diet. The reference profile is meant to represent the required profile for the nine IAAs (mg/g protein) for protein synthesis, i.e. isoleucine, leucine, lysine, aromatic amino acids, sulphur amino acids, threonine, tryptophan, valine and histidine [16]. If the AAS of all IAA > 1, AAS is truncated at 1. This truncation is needed when calculating the protein adequacy, as it corrects for the potentially limiting total amount of nitrogen [15]. The BV of the meal allows relative shortages of IAAs in one food to be compensated by IAAs in another food in the same meal. To obtain the amount of IAAs that are utilizable for protein synthesis in the body, the PDCAAS_meal_ is multiplied by the total amount of ingested protein in the meal. An example of the calculation of PDCAAS_meal_ can be found elsewhere [15] and in Online Resource 3.

The second component involves an appropriate amount of DAAs to complement the IAAs in each meal [15, 36]. Therefore, a correction of IAAs available for utilization was made when the DAAs were limiting, using the ratio of IAAs and DAAs (0.29:0.71) [16].

The third component of protein adequacy is the total daily nitrogen requirement, which is needed to assess the adequacy of protein intake. The total amount of available DAAs and IAAs for utilization on a daily basis was calculated based on Eq. (2).

2 $${\text{Total AAs }}\left( {\text{or protein}} \right){\text{available for utilization}}_{\text{day }} = { }\mathop \sum \limits_{{{\text{meal}}}} {\text{DAAs and IAAs available for utilization}}_{\text{meal}}{.}$$

To evaluate protein adequacy, this quantity is compared to the estimated average requirement (EAR) of 0.66 g/kg body weight/day for dietary protein intake across all age and gender groups in the population [14, 15]. This EAR is based on N balance studies that have estimated N balance at 105 mg/kg body weight/day. Rand et al. [17] assume that the test meals used in these studies have ‘good-quality protein’, without further specification. As the protein digestibility of especially plant-based meals is hardly ever 100% [27, 33], the PD is likely < 1. Therefore, it is assumed that Rand et al. [17] implicitly distinguish the PDCAAS into the PD and AAS and that they define good-quality protein as an AAS of 1 [17]. This is in line with studies that showed that isolated amino acids lead to greater postprandial plasma amino acid availability, while dietary proteins come in complex matrices that are not 100% digestible [37, 38]. Based on literature, we assume an average PD of 0.8 for the meals used in the studies of Rand et al. [15, 17, 27–35] . Once intake is corrected for the AAS, PD and the required ratio of IAA:DAA, the remainder is 100% utilizable and should be compared to the requirement for 100% utilizable protein. Therefore, when the hypothetical requirement for 100% utilizable amino acids is used, the requirement for protein intake amounts to 0.66 g/kg body weight/day * 0.8 = 0.53 g/kg body weight/day. This correction also holds for the IAA requirements. An example of the calculation of the protein adequacy corrected for protein digestibility and the BV is provided in Online Resource 3.

### Environmental data

GHGE (kg CO_2_ equivalents/2000 kcal) of food items was used as a proxy for the environmental impact of the diet and determined using life-cycle assessment (LCA). LCAs take into account the process of production, transportation, preparation and waste or losses of a product at all stages of the life cycle. Blonk Consultants, Gouda, the Netherlands [39] provided life cycle inventories to estimate environmental impact of a product. An extensive description of the usage of LCA in the DNFCS is described elsewhere [40].

### SHARP model

**Fig. 1:**
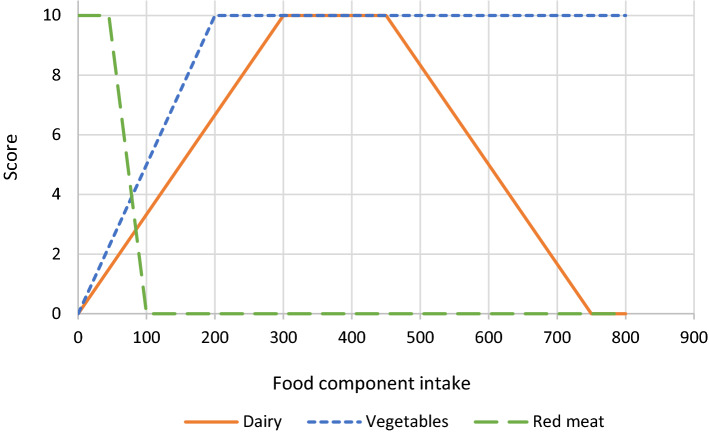
Examples of scoring components of a health indicator (dairy, vegetables and red meat) representing the score (y-axis) for each level of intake (x-axis)

To calculate healthy, sustainable and preferable diets, this study used the SHARP model which is based on nutritional benchmarking [20, 21]. Nutritional benchmarking is defined as the comparison of diets based on their nutrient (and/or food item) intakes. The SHARP model aims to calculate for each individual diet in the population a set of alternative diets that are at least as healthy as the current diet, i.e. those diets that for a certain level of less-is-better nutrients contain the highest (compared to all others) level of more-is-better nutrients or the other way around. To avoid formulating explicit acceptability constraints and imposing the current diet as a reference, the calculated diets are linear combinations of other current diets in the population. Using the current SHARP model, we can calculate healthier diets, but not the healthiest diet for an individual, because we know which nutrients should be increased or decreased, but the exact requirements were not known. In the current study, we have a score available that shows adherence to dietary guidelines, i.e. a healthy diet indicator, and thus we know the exact required consumption level of each food component. Therefore, this study extended the SHARP model by incorporating a healthy diet indicator to maximize directly on the health score, i.e. calculating the healthiest diet. As healthy diet indicators are often non-linear and based on partial scores of individual food components [23, 41, 42], the partial scores are piecewise linear functions. This implies that change of the partial score (slope) depends on the intake level. Figure 1 shows three examples of scoring components within a health indicator. The partial scores are summed up to calculate the diet’s overall health score.

The mathematical formulation of the scoring functions is provided in Online Resource 4.

### Application of the SHARP model

#### Health indicator

The DHD15 score was used as the health indicator of the diet [23]. This score was included as an objective function within the diet model as described before. This index distinguishes 15 partial scoring components, each representing 1 of the 15 Dutch dietary guidelines of 2015 [22]. A score between 0, indicating no adherence, and 10, indicating complete adherence, was attributed to each component. Since there was no information available on the type of coffee consumed (filtered or not), this item was not taken into account. DHD15 scores in this study could therefore potentially range between 0 and 140 points, where a score of 140 indicates maximal adherence to the guidelines. Cutoff values of each component are provided in Online Resource 5.

#### Phases of optimization and trade-offs

**Fig. 2:**
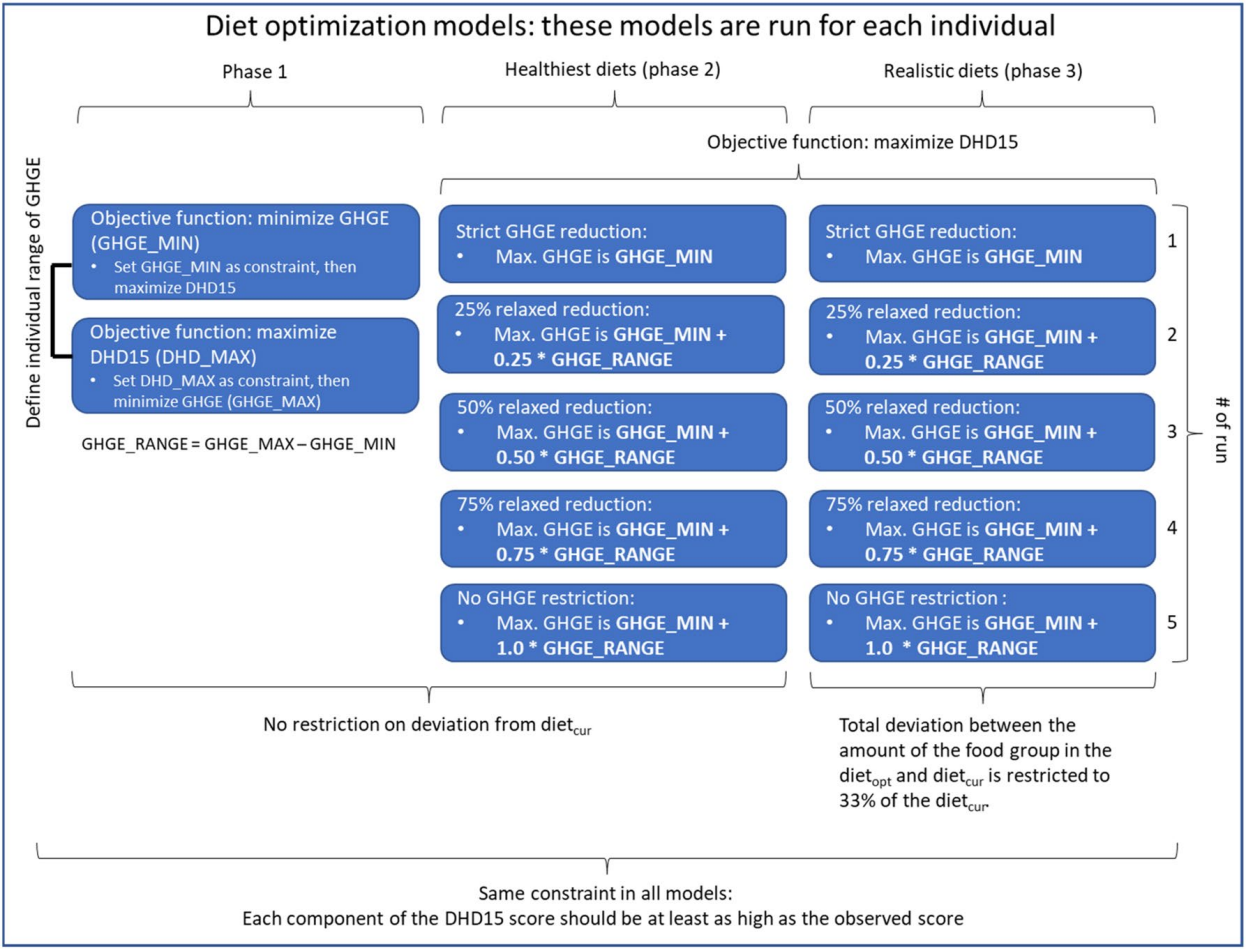
Overview of the optimization models used in this study. *GHGE* greenhouse gas emission, *DHD15* Dutch healthy diet score 2015, *Diet*_*cur*_ current diet, *Diet*_*opt*_ optimized diet

Diet optimization was performed using Xpress-IVE Version 1.25.06. Trade-offs were made between minimizing GHGE and maximizing the DHD15 using the ***ε-***constraint method [43]. The optimization process consisted of three phases (Fig. 2). In phase 1, the potential range of values for GHGE was assessed for each individual. The GHGE of the health-optimized diet (GHGE_MAX) and the GHGE-optimized diet (GHGE_MIN) created an individual range for the trade-off analyses (phase 2 and 3). In phase 2, health-optimized diets were calculated for varying constraint levels of GHGE, within the individual range of GHGE values, i.e. GHGE_MIN to GHGE_MAX. In each subsequent run, a stepwise increase of the GHGE constraint was added until the maximum GHGE was reached (5 runs in total) (Fig. 2). This way, trade-offs were made between dietary health and environmental impact. Diets resulting from phase 2 are called the ‘healthiest diets’. In phase 3, a preference constraint was added to ensure acceptability of the optimized diets, i.e. the amount in each optimized food group could deviate a maximum 33% from the mean current amount of consumers (i.e. intake > 0) in that food group. This is because the mean amount among consumers represents a realistic daily portion size [44], and deviating by one-third of a portion size is arbitrarily considered an acceptable mean change. Diets resulting from phase 3 are called ‘realistic diets’.

The two non-consecutive 24 h recalls were considered as individual diets, as averaging one’s two 24 h recalls would bias the protein adequacy. Diet optimization was performed separately for men aged 18–50 years, men aged 50–79 years, women aged 18–50 years and women aged 50–79 years to create groups of peers. The splitting of age groups was based on the menopausal timing for women, and for men similar age groups were used. As a sensitivity analysis, we split the total population by meat quartiles for current protein adequacy, protein adequacy of optimized diets and food group consumption of optimized diets. Meat quartiles were based on an individual’s current daily consumption in g/2000 kcal. Means per meat quartile per age and gender group are provided in Online Resource 6.

## Results

### Protein adequacy of current diets

**Fig. 3:**
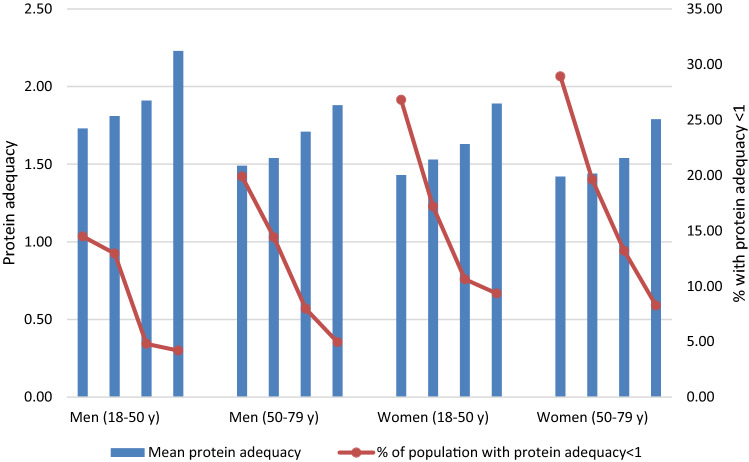
Mean current protein adequacy and the percentage of the population with inadequate protein intake (< 1) by age, gender and meat quartiles. Quartiles correspond roughly to a consumption of 9, 55, 98, and 184 g meat per 2000 kcal (Online Resource 6). Meat includes beef, pork, poultry, combined and other meat and cold cuts. The lines between the dots are meant to recognize the pattern, they are not linear interpolations

As the protein transition is motivated by supposedly adverse associations of meat consumption with health and the environment, Fig. 3 specifies protein adequacy by quartiles of meat consumption in the current diet. For men and women in both age groups, the mean protein intake was more than their protein requirements and ranged from 1.42 (meat quartile 1, women aged > 50) to 2.23 (meat quartile 4, men aged 18–50). Besides, protein adequacy increased over the meat quartiles by 0.37 (women > 50 years) up to 0.50 (men aged 18–50). The percentage of subjects with inadequate protein intake ranged from 28.9% to 4.2% (meat quartile 1, women aged 18–50, and meat quartile 4, men 18–50 years, respectively). The prevalence of inadequate protein intake decreased over the meat quartiles for each age and gender subgroup (Fig. 3).

### Protein quality of main meals in current diets

**Fig. 4:**
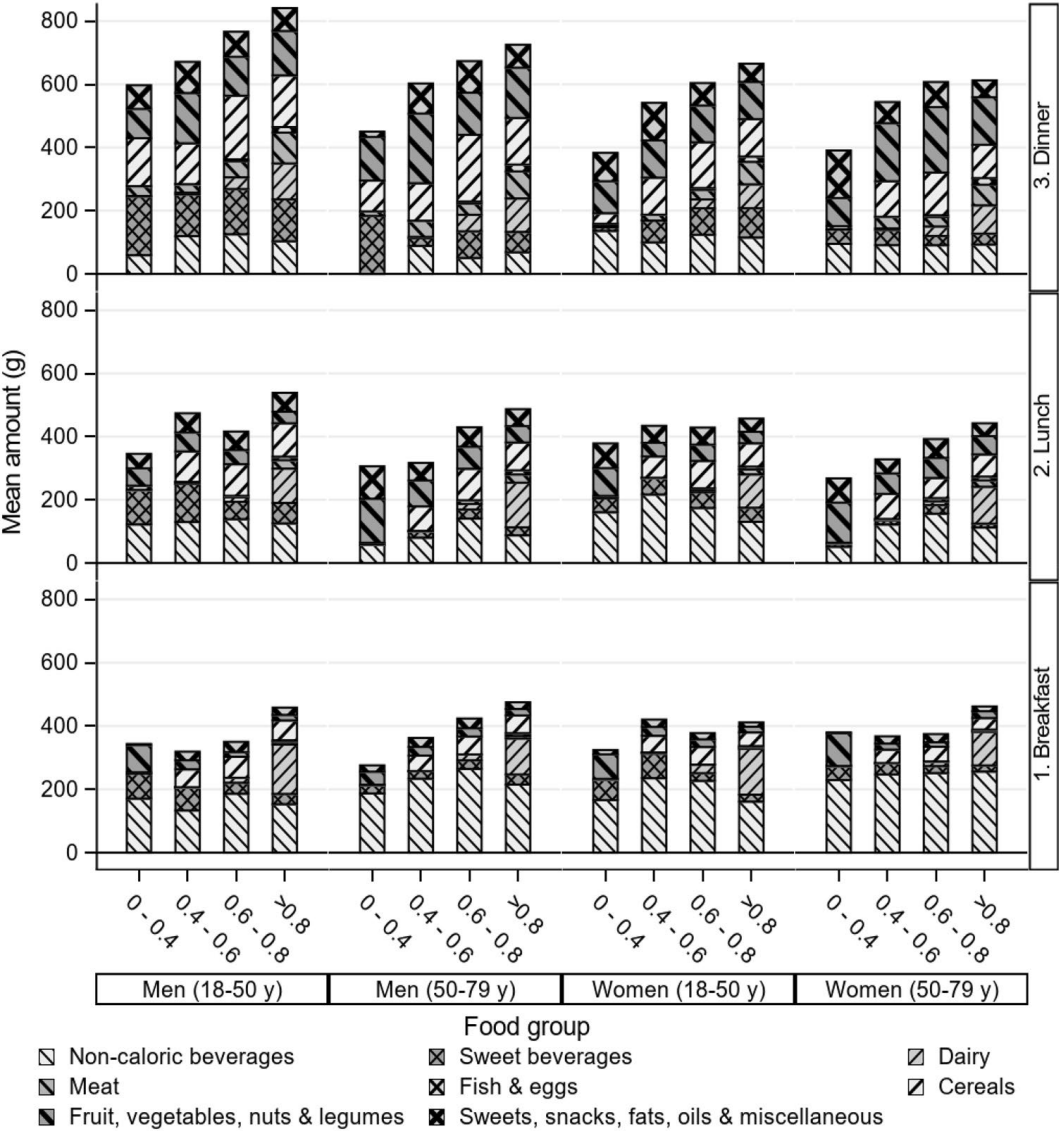
Mean consumption (gram) of food groups by protein quality (i.e. PDCAAS_meal_) and meal moment (excluding snacks in between meals), stratified for age and gender. Meat includes beef, pork, poultry, combined and other meat and cold cuts

Protein quality (PDCAAS_meal_) is associated with the amount of animal-based food groups in that meal in each age and gender subgroup. During breakfast, lower PDCAAS was associated with consumption of sweet beverages, fruit, vegetables, nuts and legumes (Fig. 4). At higher PDCAAS, these food groups were partly substituted by dairy and cereals. During lunch, higher protein quality was associated with higher consumption of dairy and lower consumption of fruit, vegetables, nuts and legumes. During dinner, higher protein quality was associated with higher consumption of meat and dairy in all age and gender groups (Fig. 4). Online resource 7 shows the average consumed digestible IAA per meal and the requirement of the corresponding IAA (both the original requirement as well as corrected for digestibility) for each age and gender subgroup. The figures show that the average consumed digestible IAAs during the main meals (breakfast, lunch and dinner) met the requirements corrected for digestibility. In between the main meals, the consumed digestible IAAs mostly did not meet the requirements.

### DHD15 score and GHGE of current and optimized diets

**Fig. 5:**
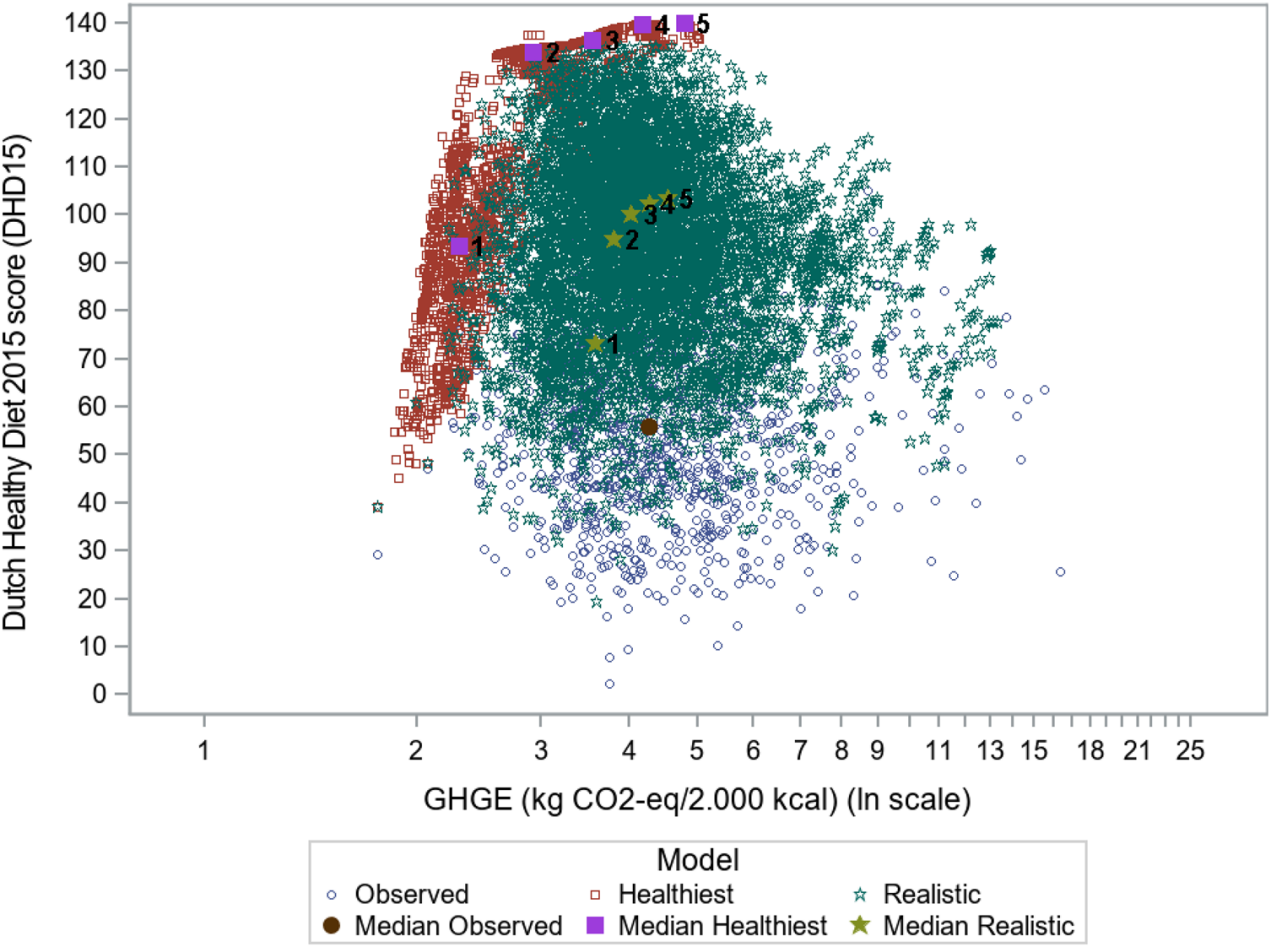
Trade-off between maximizing dietary health (DHD15) and minimizing GHGE among Dutch men (18–50 years). Filled symbols represent the medians for all ten models (‘healthiest’ (phase 2) and ‘realistic’ (phase 3), with 5 runs each) and for the current diet. Open symbols represent individual data of the current and modelled diets. ‘Healthiest diets’ have no restriction on deviation to the current diet. In ‘realistic diets’, deviation is restricted to 33% of current consumption per food group. Other age and gender strata are presented in Online Resource 8

Figure 5 shows the trade-off between diets maximized on the DHD15 score and minimized on GHGE, among men aged 18–50 years. Results for other population groups were almost similar (Online Resource 8). For the current diet, the DHD15 score and GHGE were 56 and 4.3 kg CO_2_-eq/2000 kcal. When the health score was optimized without restrictions on GHGE, the DHD15 increased to 103 for the ‘realistic diets’ (phase 3, run 5) and to its maximum of 140 when no deviance constraint was applied (phase 2, run 5). This optimization for DHD15 did not lead to reduction of GHGE, but increased GHGE to 4.5 (+ 4.7%) (realistic diets) or even 4.8 (+ 11.6%) (healthiest diets) kg CO_2_-eq/2000 kcal. When increasingly stricter criteria for GHGE reduction were applied, the DHD15 remained high until 75% of one’s maximal GHGE reduction was realized (phase 2 and 3, run 2). At this point, average GHGE was reduced to 3.8 (− 11.6%) and 2.9 (− 32.6%) kg CO_2_-eq/2000 kcal for the ‘realistic’ and the ‘healthiest diets’, respectively. Reduction of GHGE to one’s personal minimum (phase 2 and 3, run 1) strongly reduced the DHD15 to 73 (realistic diets) and 93 (healthiest diets), with only a limited further reduction of GHGE to 3.6 (− 16.3%) and 2.3 (− 46.5%) kg CO_2_-eq/2000 kcal, respectively. Yet, the DHD15-score was higher than that in the current diets (Fig. 5).

### Protein adequacy: stratification by run and quartile of meat consumption

**Fig. 6:**
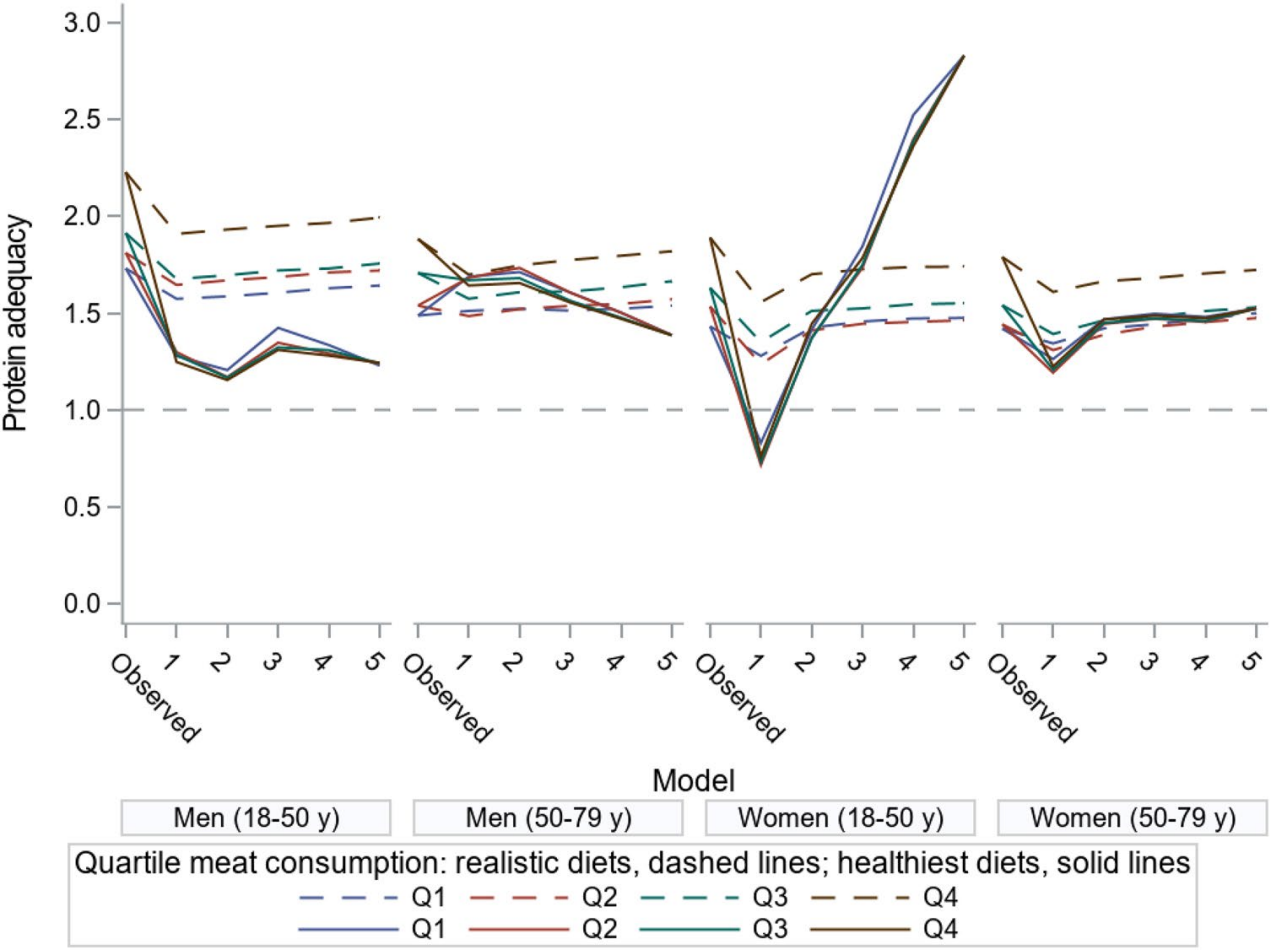
Mean protein adequacy (ratio intake versus requirements) of current and optimized diets of the Dutch population. ‘Healthiest diets’ are phase 2 models, i.e. no restriction on deviation from the current diet. ‘Realistic diets’ are phase 3 models, i.e. deviation restricted to 33% of the current consumption per food group. The lines are meant to recognize the pattern and they are not linear interpolations

Figure 6 shows how protein adequacy of the diet is affected when dietary health (DHD15) is maximized and GHGE is minimized, both with and without deviance constraints (i.e. a deviance restriction of 33% of current consumption per food group, or no deviance restriction) and in quartiles of current meat intake. The results for protein quality show considerably more variability within the population subgroups below age 50 as compared to older men and women in the ‘healthiest diets’, i.e. no restriction on deviation to the current diet. Besides, the protein adequacy was almost similar in all meat quartiles. Among men, the adequacy ratio was 1.2–1.4 for young men and 1.4–1.7 for older men, i.e. less than for current diets (1.7 to 2.2 for young men and 1.4–1.9 for older men). Among women below age 50, the adequacy ratio is lowest (0.7) in the ‘healthiest diets’ where GHGE is minimized (run 1) and increases to 2.8 when GHGE constraints are successively relaxed (run 1–5). The pattern in women above age 50 is similar, though less extreme (increase from 1.2 to 1.5). For ‘realistic diets’, where deviation is restricted to 33% of the current consumption per food group, these patterns are very much attenuated for all four population subgroups. Protein quality is highest in current diets and decreases when GHGE is further minimized, with the adequacy ratio being lowest (1.2) for women up to age 50. In summary, in the ‘realistic diets’ for both men and women, there is considerable room for simultaneously increasing the DHD15 score and reducing the GHGE footprint with only small sacrifices of the protein adequacy, and the mean protein intake remains adequate. However, for the ‘healthiest diets’ minimizing GHGE to the lowest possible value could raise concerns on protein quality for younger women. This was because the low protein diet of one woman was chosen by the model as a peer for most other diets. This woman mainly consumed water, tea and some sweets, providing a very low GHGE. Overall, there is a tendency toward higher protein quality at higher meat quartiles for all age and gender subgroups, especially in the current and ‘realistic diets’ (Fig. 6).

### Food intake of current and modelled diets

**Fig. 7:**
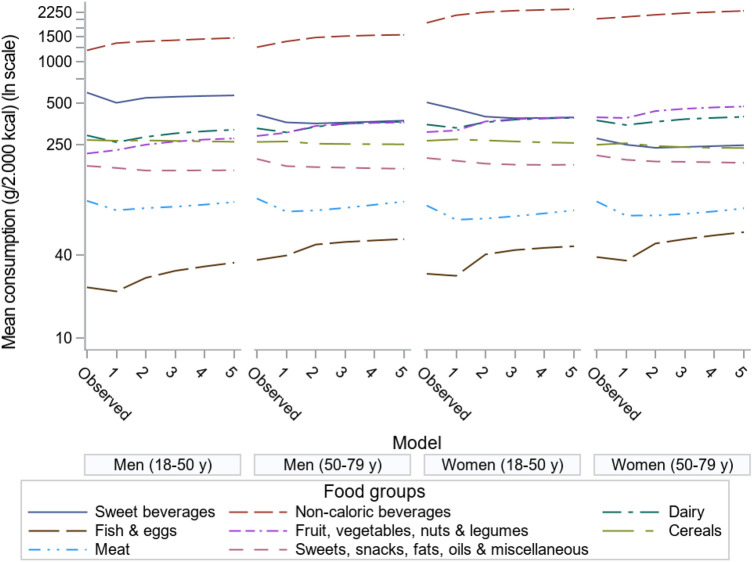
Mean food group consumption in g/2000 kcal by current diets and ‘realistic diets’, i.e. deviation restricted to 33% of current consumption per food group, per subgroup of age and gender. The lines in between are meant to recognize the pattern and they are not linear interpolations

Figure 7 summarizes the mean intake of food groups for current and ‘realistic diets’, i.e. the amount in each optimized food group could deviate by maximum 33% from the mean current amount of consumers (i.e. intake > 0) in that food group in the corresponding population subgroup. For ‘realistic diets’, the GHGE-minimized diets (run 1) contained higher amounts of plant foods such as fruits, vegetables, nuts and legumes compared to current diets, which increased even more when GHGE constraints were relaxed to meet optimal healthy diets (run 5). Note that diets of older women had little room for improvement, as their current diets already contained more plant foods than the other population subgroups. Regarding animal-sourced foods, the GHGE-minimized diet (run 1) contained less fish and eggs compared to the current diet (except for older men), but consumption was higher when the GHGE constraint was relaxed and health was maximized (run 5). A similar, but weaker tendency is visible for meat and dairy products. Compared to current diets, the amount of sweets, snacks, fat and oils was reduced in all modelled diets, whereas cereal products remained almost similar for current and GHGE-reduced diets. Regarding drinks (milk was grouped as dairy), the optimized diets contained more non-caloric beverages (i.e. water, coffee and tea) and less sweet beverages (i.e. soft drinks and fruit juice) compared to current diets. This substitution was most clear when GHGE was restricted most (run 1 and 2) (Fig. 7).

Online Resource 9 shows the food group consumption of ‘realistic diets’ per meat quartile. The major difference compared to that in Fig. 7 is the exchange of meat, for fish and eggs when moving from the lower to the higher meat quartiles in each age and gender subgroup (Online Resource 9).

### Further characteristics of the diet

Online Resource 10 shows some nutrients of the current diet and of run 2 of the ‘realistic diets’, i.e. the diet that is on the trade-off. The energy percentage protein is about similar between the current and the optimized diets for each age and gender subgroup. The percentage plant protein is higher in the optimized diet (55–36% for younger men, 49–35% for older men, 54–36% for younger women and 47–34% for older women, ranges from low to high meat quartiles) than in the current diet (55–31% for younger men, 48–30% for older men, 53–30% for younger women and 46–30% for older women). The difference in percentage plant protein between the optimized and the current diet increases with increasing meat quartiles. For older women, it is striking that vitamin B12 is higher in the optimized diet (from the second quartile of meat consumption onwards) compared to the current diet, while the percentage plant protein is also higher in the optimized diets. Calcium and iron levels do not differ a lot between the current and the optimized diets and are all above their EARs.

## Discussion

This study shows that the mean current intake of utilizable protein in the Dutch dietary pattern is more than the estimated average requirement. Current protein quality is highest when animal-based products are consumed within a meal. The modelled diets showed a clear trade-off between the DHD15 diet quality score and GHGE of diets. For both the ‘healthiest’ and the ‘realistic diets’, the largest improvement of the DHD15 score was reached when moving from maximum GHGE reduction to 75% reduction of one’s individual GHGE range, with protein adequacy still above the adequate level. For the ‘realistic diets’ (deviation restricted to 33% of current consumption per food group) with GHGE reduced to 75% of one’s individual range, the median DHD15 score almost doubled and mean protein quality was only slightly lower than that for the current diets. When relaxing the deviance constraint, i.e. prioritizing ‘healthiest diets’ above ‘realistic diets’, DHD15 scores reached higher levels and GHGE reached lower levels in the situation of 75% GHGE reduction of one’s individual range, showing a trade-off between preferability and acceptability versus health and sustainability of diets.

### Protein quality

Evaluation of protein quality requires a meal-based analysis, rather than a daily-based approach, as amino acids within a meal can complement each other [15]. Therefore, calculation of protein intake should ideally take into account combinations of products, protein digestibility and amino acid scores within meals, using meal-based PDCAAS or DIAAS. As our optimization model makes linear combinations of days, meal composition of the selected days was not affected. Therefore, the weights of each selected day could be interpreted as frequencies by which such days should occur in an optimized diet.

‘Realistic diets’ (phase 3 models) with maximum GHGE reduction contain lower amounts of animal-based products compared to the current diet, but still have an adequate protein level on average. Less than 11% of the total population had inadequate protein intake when GHGE is minimized (data not shown). The highest percentage of inadequate protein intake was found in the lowest meat quartile (19.3%), while the lowest percentage was found in the highest meat quartile (7.0%). When moving from a low to a high meat consumption, protein quality slightly increased. The partly exchange of meat, for fish & eggs when moving from higher to lower meat quartiles may explain the protein adequacy that remained adequate over the meat quartiles. The exchange of animal products reveals that a low-meat diet does not by definition mean a more plant-based diet. In mainly plant-based diets in other Western countries, adequate intake levels of protein and amino acids were also observed [45]. However, these studies considered daily intake rather than meal moment to estimate uitilizable protein, implying that their protein adequacies may be overestimated. The percentage of protein that can be utilized was positively associated with the percentage of animal protein in the diet (data not shown) [15]. This emphasizes the need for a focus on protein quality and quantity when shifting to a more sustainable, and thereby more plant-based diet.

To assess protein quality, the current recommendation is to use the digestible indispensable amino acid score (DIAAS) instead of the PDCAAS [16]. However, data on DIAAS is only available for some ingredients and a few products [46]. In DIAAS the ileal digestibility of each IAA is determined, whereas in the PDCAAS a specific digestibility factor is determined for food(s) (groups) using faecal digestibility and the same factor is applied to each IAA. Besides, the FAO recommended that for foods susceptible to damage from processing, ‘reactive’ rather than ‘total’ lysine contents and the true ileal digestibility of reactive lysine (lysine availability) rather than of total lysine are used when calculating DIAAS scores [16]. Because of the reactivity of lysine, PDCAAS may overestimate its digestibility. Also faecal digestibility overestimates digestibility of IAA, compared to ileal digestibility [18]. The PDCAAS method can overestimate protein quality, in some cases even exceeding 10%. This holds especially for dietary protein with low protein digestibility [18]. The overestimation is due to IAA absorbed in the hindgut that is unlikely to contribute to the metabolic amino acid pool. Another limitation is that each food item within a food group obtained a similar protein digestibility factor. However, protein digestibility of different food items may differ slightly (Online Resource 1b). Furthermore, we did not take into account processing of food products when defining the protein digestibility factor. For most products of which protein digestibility after processing was measured, digestibility increased [18], which may lead to an underestimation of the protein adequacy as calculated in the current study.

### Modelling

Several studies performed diet optimization techniques based on combining food items [19, 47, 48]. For example, the Dutch diet was optimized based on quadratic optimization of food items, by minimizing the deviance from the average baseline diet while satisfying nutritional goals and remaining below GHGE targets [19]. They found that a shift away from beef, cheese, butter and snacks was required towards plant-based foods and fish and shellfish. In the vegan scenario in this study, 532 g of soy drink was suggested by the model, while in the current diet only 9 g was consumed, questioning acceptability of the calculated diet. To account for acceptability, food item-based models need constraints based on expert opinion to preserve implicit associations and interdependent quantities of food items [11, 19, 49]. Furthermore, in food item-based models, uncertainties in the data can have a large influence on the outcome. Therefore, several studies based their optimization on linear combinations of food groups instead of food items [49, 50]. However, food grouping reduces the detail of the data and remains an arbitrary process.

To circumvent data uncertainties, arbitrary food grouping within the modelling and acceptability constraints based on expert opinions, a diet modelling study in four European countries calculated optimized diets by making linear combinations of complete diets rather than food items [21]. Diets were optimized on either preferability, nutrient content (Nutrient Rich Diet score, NRD15.3) or GHGE. This way, basic interrelationships among food items are implicitly maintained. In this study, diets were benchmarked by identifying those diets that have a higher ratio of more-is-better ‘healthy’ nutrient content per unit of less-is-better ‘unhealthy’ nutrients, obtaining a set of healthier diets. This set of healthier diets was then optimized for preferability, nutrient content or GHGE. Diets optimized on nutrient quality exhibited the highest amounts of plant-sourced foods, while diets optimized for GHGE contained the lowest amounts of red and processed meat. Because optimized diets are weighted averages of existing diets, they stay within the range of current diets. Therefore, this type of model does not obtain the theoretical optimal diet from a strict health or environmental view only. However, realistic stepwise changes provide a first step in the shift towards more healthy and sustainable diets, compared to the theoretical but unrealistic optimal diet.

In a Japanese study, a similar benchmarking method was used, but now diets were maximized on cultural acceptability and the Nutrient-Rich Food Index 15.3 score and minimized on monetary cost and diet-related GHGE [51], rather than optimizing on one of these objectives as Mertens et al. [21] did. Because of the trade-offs, improvements of the diets were at a moderate level. Dietary intake patterns demanded increases in consumption of whole grains, legumes, nuts, seeds, fruits and dairy, and decreases in consumption of red and processed meat, sugar and confectioneries, alcoholic and sweetened beverages and seasonings. Rather than benchmarking diets based on less-is-better nutrients and more-is-better nutrients, in the present study we extended the SHARP model to be able to benchmark diets by maximizing a healthy diet (DHD15) score. Because of this, we were able to find the ‘healthiest’ diet instead of ‘healthier’ diets, as done by Mertens et al. and Sugimoto et al. [21, 51]. Like Mertens et al. [21] did for other EU countries, and Sugimoto et al. 2022 for Japan, our study also showed the health–environment trade-off. In addition, we added the trade-off of these two diet outcomes against acceptability of the diets, by comparing the ‘healthiest diet’ models with ‘realistic diet’ models.

As a next step, following on the required meal-based analysis for protein quality, a meal-based approach might also be an attractive option to model healthy and sustainable diets, by optimizing frequencies of healthy and sustainable meals over a reference period of e.g. 1 week. Food choices are guided by geographics and socially determined taste, texture and price of meals [52]. Eating habits directly affect health via eating rate and protein quality and affect nutritional quality and the environment in the long run [53]. Such a meal-based approach would also account for cultural and sensory issues, which may increase the acceptability of diets by consumers.

### Trade-offs

Our results emphasize the trade-off between public health and environmental sustainability. In the ‘realistic diets’ (phase 3), when GHGE is minimized (run 1), the median GHGE already exceeds 3.5 kg CO_2_-eq/2000 kcal (Fig. 5, Online Resource 8). For the ‘healthiest diets’ (phase 2) the minimum GHGE is 2.0 kg CO_2_-eq/2000 kcal (Online Resource 8), which is not within the planetary boundaries of 2.04 or 1.11 kg CO_2_-eq/day for 2030 and 2050, respectively [19]. Thus, fully optimizing for health, e.g. according to the nutritional guidelines [22] would not be compatible with the planetary boundaries for food production and consumption. However, a first step can be taken in reducing GHGE and increasing dietary health, for example, by aiming for a 75% reduction of one’s possible range of GHGE, while reaching relatively much health gain: 12–16% reduction of GHGE to 3.8–4.3 kg CO_2_-eq/day, 60–75% increase in DHD15 score to 95–110 (out of 140), compared to the current diet. Thus, behaviour change is necessary, but large behaviour changes may not be realistic and will not be sufficient, which stresses the need for a fundamental food systems change, including more sustainable agricultural practices, adapted supply chains and new healthy and sustainable food products [54–56]. Nevertheless, when considering the consumption perspective, our model points at the direction of realistic changes that might be feasible for consumers in this decade.

To conclude, the average current Dutch diet of adults contains an adequate amount of utilizable protein. When optimizing ‘realistic diets’ on health and environmental sustainability, a clear trade-off between these two outcomes was apparent. The most healthy diets had highest GHGE, also higher than current diets, and the most sustainable diets had lowest dietary quality, tough still higher than current diets. When keeping changes of consumption per food group within 33% of the current amounts, a realistic aim for the next decade might be to reduce GHGE by 75% from one’s potential, which corresponds to a 12–16% reduction of GHGE for the population, and keeps protein adequacy above the adequate level. Further improvements in dietary health and environment would need fundamental changes in agriculture, food processing and the food environment of consumers.

## Supplementary Information

Below is the link to the electronic supplementary material.

Supplementary file1 (DOCX 1472 KB)

## Data Availability

Data of the Dutch National Food Consumption Survey 2012–2016 can be requested for at https://www.rivm.nl/en/dutch-national-food-consumption-survey/data-on-request (Accessed on 16 October 2019). Primary environmental data of 250 food products can be found at https://www.rivm.nl/voedsel-en-voeding/duurzaam-voedsel/databasemilieubelasting-voedingsmiddelen (Accessed on 16 October 2019).
