# Supplementary material for: Shifting towards optimized healthy and sustainable Dutch diets: impact on protein quality
Source: Eur J Nutr. 2023 Mar 23;62(5):2115–28. doi: 10.1007/s00394-023-03135-7 (PMC10349729; doi:10.1007/s00394-023-03135-7)
Supplement: Supplementary file 1 — Supplementary file1 (DOCX 1472 KB) [file 394_2023_3135_MOESM1_ESM.docx]

**Title:**

Shifting towards optimized healthy and sustainable Dutch diets: impact on protein quality

**Journal:**

European Journal of Nutrition

**Authors:**

Samantha N. Heerschop^a^, Argyris Kanellopoulos^b^, Sander Biesbroek^a^, Pieter van ‘t Veer^,a^.

a: Division of Human Nutrition and Health, Wageningen University & Research, Wageningen, Gelderland, The Netherlands;

b: Operations Research and Logistics Group, Wageningen University & Research, Wageningen, Gelderland, The Netherlands;

**Corresponding author:**

Samantha Heerschop

[samantha.heerschop@wur.nl](mailto:samantha.heerschop@wur.nl)

Postal address: Division of Human Nutrition and Health, postbox 17, 6700 AA Wageningen

# Online Resource 1

## Procedure to add amino acids to the Dutch Food Composition Table

1. Products of the Dutch Food Composition Table (NEVO)—consumed in the Dutch National Food Consumption Survey 2012-2016—containing more than 1 gram protein per 100 gram product were linked to similar products in the Danish Food Composition Table (Frida).
   1. The Frida table was chosen as their products were deemed similar to Dutch products. Additionally, their database contains amino acid values for 799 products compared to 690 products in the Mccance and Widdowson (English database).
   2. All amino acids for products containing <1 gram protein per 100 gram product were set to zero.
   3. The following amino acids were added to NEVO:
      1. Isoleucine
      2. Leucine
      3. Lysine
      4. Methionine
      5. Cystine
      6. Phenylalanine
      7. Tyrosine
      8. Threonine
      9. Tryptophan
      10. Valine
      11. Arginine
      12. Histidine
      13. Alanine
      14. Aspartic acid
      15. Glutamic acid
      16. Glycine
      17. Proline
      18. Serine
2. For Dutch products without a similar product equivalency in Frida, the following methods were applied: a) either a ‘basic’ product was chosen based on the largest protein source of the product; b) a comparable product was chosen to obtain the amino acid profile; c) other databases were accessed to obtain the amino acid information; d) a recipe was created; e) or, specific data were obtained from Friesland Campina for certain dairy products.
   1. Basic product - For example, a ’doughnut plain’ in NEVO was linked to ‘wheat flour’ in Frida given that wheat flour is the main protein-containing ingredient in a doughnut.
   2. Comparable product – For example, if fruit yogurt was not available in Frida, the amino acid profile of plain yogurt was used instead. Specifically for some types of fish, another fish was chosen that did have amino acids available in Frida.
   3. Other databases - For instance, the USDA, Mccance & Widdowson, and the Japanese food composition table were utilized. The choice between these databases was made based on the availability of products and their corresponding amino acid data as well as their comparability to the Dutch product.
   4. Recipe - If the product in NEVO consisted of several protein sources, a recipe was created from several products existing in NEVO. In this recipe, it was ensured that the protein content was similar to the NEVO product. If the protein content was reached by a percentage of ingredients less than 100%, water was used in the remainder of the recipe to reach 100%. As such, these recipes can only be used for the purpose of this amino acid table. Recipes created for this amino acid table are indicated with ‘extra recipe’ in columns ‘remarks’ or ‘source’.
   5. Friesland Campina - The Dutch Dairy Company ‘Friesland Campina’ provided us with analyzed amino acid values specifically for quark.
3. When all products were linked, the amino acid profiles were adjusted for the amount of protein that was present in Dutch products according to the following calculation:

$$mg amino acid in NEVO=\frac{gram protein in NEVO * mg amino acid in Frida}{gram protein in Frida}$$

4. The adjusted amino acid profiles were merged with the NEVO table in SAS.
5. The amino acid values of recipes were calculated by running a script in R. In the first step of the script all recipes were broken down into single ingredients. In the second step, adjusted amino acid profiles and protein per 100g from step 4 were linked to the single ingredients within the recipes. The amino acid profile and protein per 100g were then multiplied by the proportion per ingredient. Per recipe the sum per amino acid was taken to calculate the total AZ profile per recipe. Next, the amino acid profiles were adjusted for the amount of protein in NEVO according to the following calculation:

$$mg amino acid in NEVO=\frac{gram protein in NEVO * mg amino acids in recipe}{gram protein in recipe}$$

Finally, the adjusted amino acid profiles were merged with NEVO in R.

This dataset is available from the authors on reasonable request.

## b. Linking digestibility factors to NEVO

To be able to calculate PDCAAS, each product in the NEVO table was assigned a digestibility factor. The procedure was as follows:

1. Fecal digestibility factors were assigned to NEVO food groups. These digestibility factors were then automatically assigned to all NEVO foods within their respective NEVO food group.
2. To define the digestibility factor per food group a literature search was performed to get an overview of digestibility factors of different foods within each NEVO food group. Digestibility factors were obtained from several sources. Based on comparability of food items within a food group a digestibility factor for the respective NEVO food group was chosen for all foods within a food group. Important to note is that a separate digestibility factor was chosen for plant protein and animal protein sources within a NEVO food group. For plant-based foods, the average of the digestibility factor of these foods was chosen as the digestibility factor. The same approach was applied for animal-based foods. For mixed foods, the digestibility factor of the animal-based food was chosen, as the animal-based part of the product probably provides the largest share of protein.

Supplemental table 1. Digestibility factor per food group

| **NEVO product group** | **NEVO product group description** | **Overview of digestibility factors in literature per food group** | **Final digestibility factor** |
| --- | --- | --- | --- |
| 1 | Potatoes and tubers | - Potatoes: 0.55 [1] - Sweet potatoes:0.5 [1]   Potatoes are more often consumed than sweet potatoes, therefore 0.55. | 0.55 |
| 2 | Alcoholic beverages | Plant-based products | 0.65 |
| 3 | Bread | 0.9 [1] | 0.9 |
| 4 | Miscellaneous foods (plant-based foods such as seaweed, cacao powder, yeast, etc.) | Seaweed: 0.43 [2]  Yeast: 0.82 [3]  Cacao powder: 0.36 [4]  Almonds (for almond paste): 0.88 [4] | 0.65 |
| 5 | Eggs | 0.97 [5, 6] | 0.97 |
| 6 | Fruits | - Kiwi: 0.6 [1] - Fruit 0.76 [7] | 0.76 |
| 7 | Pastry and biscuits | - Biscuits: 0.9 [1] - Wheat flour biscuit: 0.9 [1] | 0.9 |
| 8 | Cereals and cereal products | - Barley: 0.78 [1] - Breakfast cereal [1]:   Flaked corn 0.67 [1]  Rolled oat 0.9 [1]  Wheat bran 0.73 [1]   - Corn/corn flour: 0.82 [1] - Oats: 0.74 [1, 4, 8] - Rice: 0.9 (but cooked 0.7) [1], 0.88 [5] - Whole wheat 0.45 [7] - Wheat: 0.93 [1], 0.86 [5] - Wheat flour: 0.9 [1] | 0.7 |
| 9 | Vegetables | 0.65 [5] | 0.65 |
| 10 | Savory bread spreads | - Peanut butter 0.95 [6] - Sandwich spread (vegetable spread): 0.65 [5]   Sandwich spread is only a small part of the products in this group | 0.9 |
| 11 | Savory sauces |  | Animal-based: 0.9  Plant-based: 0.65 |
| 12 | Savory snacks | - Potato crisps: 0.47 [1] - Potato fries: 0.50 [1] | Animal-based: 0.9  Plant-based: 0.65 |
| 13 | Cheese | 0.95 [5, 6] | 0.95 |
| 14 | Herbs and spices | Plant-based products | 0.65 |
| 15 | Milk and milk products | Milk: 0.95 [1, 5] | 0.95 |
| 16 | Non-alcoholic beverages | Plant-based products | 0.65 |
| 17 | Nuts and seeds | - Linseed: 0.74 [1] - Sun flour seed: 0.78 [1] - Peanut: 0.9 (roasted) [1] - Hemp seed 0.66 [4] | 0.75 |
| 18 | Legumes | - Peas: 0.78 [1] - Pea 0.7 [1] - Kidney beans: 0.78 [1] - Mung beans: 0.82 [1] - Soyabeans: 0.68 (cooked) [1] - Field beans: 0.75 [1] - Jack beans: 0.6 [1] - Chickpeas 0.7/0.8 [9] - Kidney beans 0.74[1] - Black beans 0.5/ 0.75 [4] - Lentils 0.8 [1, 4] - Beans 0.78 [6] | 0.75 |
| 19 | Clinical formulas (Foods for special nutritional value) | Infant formula: 0.9 (goat and cow milk based) [1] | 0.9 |
| 20 | Mixed dishes | Mix of animal and plant-based products | Animal-based: 0.9  Plant-based: 0.65  Mix of animal and plant--> take 0.9 |
| 21 | Soups | Mix of animal and plant-based products | Animal-based: 0.9  Plant-based: 0.65  Mix of animal and plant--> take 0.9 |
| 22 | Sugar, sweets and sweet sauces | Mix of animal and plant-based ingredients therefore in between 0.65 and 0.9 | 0.8 |
| 23 | Fats and oils | Plant-based products | 0.65 |
| 24 | Fish | 0.9 [1]  0.94 [5, 6] | 0.9 |
| 25 | Meat and poultry | - Beef 0.92 [7] - Meat: 1 [8] - Poultry 0.95 [8] | 0.95 |
| 26 | Meat substitutes and dairy substitutes | - Soy 0.91 [5] - Soya 0.94 [1] - Soy 0.95-0.98 [10] | 0.94 |
| 27 | Cold meat cuts | Considered similar as meat and poultry | 0.95 |

## References

1. Gilani S, Tomé D, Moughan P, and Burlingame B, *The assessment of amino acid digestibility in foods for humans and including a collation of published ileal amino acid digestibility data for human foods: Report of a Sub-Committee of the 2011 FAO Consultation on “Protein Quality Evaluation in Human Nutrition”*. 2011.

2. Cian RE, Fajardo MA, Alaiz M, Vioque J, et al. (2014). Chemical composition, nutritional and antioxidant properties of the red edible seaweed Porphyra columbina. *International journal of food sciences and nutrition* **65**(3): 299-305. DOI: 10.3109/09637486.2013.854746.

3. Marques F, Lasanta C, Caro I, and Pérez L (2008). Study of the lipidic and proteic composition of an industrial filmogenic yeast with applications as a nutritional supplement. *Journal of agricultural and food chemistry* **56**(24): 12025-12030. DOI: <https://doi.org/10.1021/jf802040k>.

4. Hohnstein J, *Protein Digestibility*. 2019.

5. Tome D (2012). Criteria and markers for protein quality assessment–a review. *British Journal of Nutrition* **108**(S2): S222-S229. DOI: <https://doi.org/10.1017/S0007114512002565>.

6. World Health Organization. (2007). Protein and amino acid requirements in human nutrition. *World health organization technical report series*(935): 1.

7. Lanham-New SA, Stear SJ, Shirreffs SM, and Collins AL, *Sport and exercise nutrition*. 2011: Wiley-Blackwell.

8. Ciuris C, Lynch HM, Wharton C, and Johnston CS (2019). A comparison of dietary protein digestibility, based on DIAAS scoring, in vegetarian and non-vegetarian athletes. *Nutrients* **11**(12). DOI: 10.3390/nu11123016.

9. Food and agriculture organization of the United Nations and World Health Organisation., *Protein quality evaluation: Report of the Joint FAO/WHO Expert Consultation*. 1991: Rome, Italy.

10. Rizzo G and Baroni L (2018). Soy, soy foods and their role in vegetarian diets. *Nutrients* **10**(1): 43. DOI: 10.3390/nu10010043.

# Online Resource 2

Supplemental table 2. Categorization of 28 food groups adapted from GloboDiet,based on the Dutch National Food Consumption Survey 2012-2016.

| **Food group** | **Food subgroup** | **GloboDiet groups** | **Explanation type of product within food group** |
| --- | --- | --- | --- |
| Animal based foods | | | |
| Meat | Beef | ’07-01-01’ Beef |  |
|  | Pork | ’07-01-03’ Pork |  |
|  | Poultry | ’07-02-01’ Chicken, hen  ’07-02-02’ Turkey, young turkey |  |
|  | Combined and other meat | '07-00' Unclassified and combined meat and meat products  '07-01-00' Unclassified, mixed and other mammals  '07-01-02' Veal  '07-01-04' Mutton/lamb  '07-01-05' Horse  '07-01-06' Goat  '07-01-07' Rabbit  '07-02-00' Unclassified and other poultry  '07-02-03' Duck  '07-03' Game  '07-04-00’ Unclassified processed meat  '07-04-01’ Hot processed meat  '07-05' Offals |  |
|  | Cold cuts | '07-04-02’ Cold processed meat |  |
|  | Meat replacer | ’07-06’ Meat substitutes  ’17-01’ Vegetarian products/dishes | Unclassified meat substitutes  Hot meat substitutes  Cold meat substitutes |
| Dairy | Dairy | ’05-00’ Unclassified and mixed dairy products  ’05-01’ Milk, milk beverages and fermented milk beverages  ’05-03’ Yogurt  ’05-04’ Fromage blanc, petits suisses (quark)  ’05-06’ Cream desserts, puddings (milk based)  ’05-07-00’ Unclassified creams  ’05-07-01’ Dairy creams and creamers  ’05-08-00’ Unclassified, combined ice creams/sorbets  ’05-08-01’ Ice cream (milk based) | Unclassified or combined milk and milk beverages  Non fermented milk and milk beverages  Fermented milk, milk beverages and yogurt drinks |
|  | Cheese | ’05-05’ Cheeses (including spread cheeses) |  |
|  | Dairy replacers | ‘05-02’ Milk substitutes and milk substitute products  ‘05-07-02’ Non-dairy creams and creamers  ’05-08-02’ Ice cream substitutes  ’05-08-03’ Sorbet/water ice |  |
| Fish & eggs | Fish | ‘08’ Fish, shellfish and amphibians | Unclassified and combined fish products,  Fish  Crustaceans, mollusks  Fish products, fish in crumbs  Amphibians and reptiles |
|  | Eggs | ‘09’ Eggs and egg products |  |
| Plant-based foods | | | |
| Cereals | Potatoes | ‘01’ Potatoes and other tubers |  |
|  | Refined grains ^a^ | ‘06’ Cereals and cereal products \|<25% whole grains and cereal bran | Unclassified and combined cereal products  Flours, starches, flakes, and semolina used as flour  Pasta, rice, other grain  Bread, crispbread, rusks  Breakfast cereals  Dough and pastry (plain puff, short-crust, pizza) |
|  | Whole grains ^a^ | ‘06’ Cereals and cereal products \|>25% whole grains and cereal bran | Unclassified and combined cereal products  Flours, starches, flakes, and semolina used as flour  Pasta, rice, other grain  Bread, crispbread, rusks  Breakfast cereals  Dough and pastry (plain puff, short-crust, pizza) |
| Fruit, vegetables, nuts, and legumes | Vegetables | ‘02’ Vegetables | Unclassified, mixed salad/vegetables  Leafy vegetables (except cabbages)  Fruiting vegetables  Root vegetables  Cabbages  Mushrooms  Frain and pod vegetables  Leek, onion, garlic  Stalk vegetables, sprouts |
|  | Legumes | ‘03’ Legumes |  |
|  | Fruits | ’04-01’ Fruits  ’04-03’ Olives | Unclassified, mixed fruits, fruit compote, fruits |
|  | Nuts and seeds | ’04-02’ Nuts, peanuts, seeds and nut spread  ’04-00’ Unclassified, mixed fruits, nuts and seed | Unclassified nuts and seeds (+ nut spread),  Nuts, peanuts, seeds,  Peanut butter, nut/seeds spread |
| Beverages | | | |
| Sweet beverages | Fruit and vegetable juice | ‘13-01’ Fruit and vegetable juice |  |
|  | Soft drinks | ’13-00’ Unclassified and combined non-alcoholic drinks  ‘13-02’ Carbonated/soft/isotonic drinks, diluted |  |
|  | Alcoholic beverages | ‘14’ Alcoholic beverages | Unclassified, cocktails, punches  Wine, cider, fruit wines  Fortified wines (sherry, Porto, vermouth),  Beer  Spirits, brandy  Aniseed drinks  Liqueurs |
| Non-caloric beverages | Coffee and tea | ‘13-03’ Coffee, tea and herbal tea | Unclassified and combined coffee/tea drinks  Coffee  Tea  Herbal tea  Chicory, substitutes |
|  | Water | ‘13-04’ Water |  |
|  |  |  |  |
| Miscellaneous | | | |
| Sweets, snacks, and miscellaneous | Sweets and snacks | ‘11’ Sugar and confectionery  ‘12’ Cakes and sweet biscuits | Unclassified or combined confectionery items  Unclassified and other sugar, honey, jam  Sugar  Jam, jelly, marmalade  Honey  Other sweet spread  Sweet sauce, sweet topping for desserts  Syrup (incl. from can and for beverages)  Unclassified and other chocolate confectionary (incl. sauce)  Chocolate tablet  Chocolate candy bars  Chocolate spread and chocolate powder  Chocolate confectionery  Confectionery non chocolate  Unclassified and combined cakes, biscuits  Cakes, pies, pastries, puddings (non-milk based)  Dry cakes, sweet biscuits |
|  | Salty snacks | ‘18’ Savory snacks. | Unclassified or combined snacks  Savory snacks, biscuits and crisps  Savory filled buns, croissants |
|  | Fats and oils | ‘10’ Fats and oils | Unclassified and combined fats  Vegetable oils  Butter  Margarines and cooking fats  Other animal fats (including fish oils) |
|  | Broth, sauces and condiments | ‘15’ Condiments, spices, sauces and yeast and  ‘16’ Soups and stocks | Unclassified or combined condiments and sauces  Other and mixed sauces  Tomato sauces  Dressing sauces, mayonnaises and similar  Mayonnaise based spreads  Yeast  Spices, herbs and flavorings  Unclassified and combined condiments  Vinegar |
|  | Other | ‘17’ Miscellaneous (excluding ’17-01’ Vegetarian products/dishes)  ‘19’ Ready meals | Unclassified or combined miscellaneous foods Unclassified and combined dietetic products  Artificial sweeteners  Meal substitutes  Insects  Unclassified ready meals  Jarred meals for children |

^a^ Grouping into refined or whole grains was done based on the cut-off value of the Dutch dietary guidelines: cereals and cereal products that exist of at least 25% whole grains and cereal bran [1]. The script is available on request from the authors.

## References

1. Dutch Health Counsil., *Cereals and cereal products - Background Document for Dutch Dietary Guidelines 2015; Granen en graanproducten - Achtergronddocument bij Richtlijnen goede voeding 2015*. 2015, Dutch Health Counsil: The Hague, The Netherlands.

# Online Resource 3

Supplemental table 3. Example of the calculation of a PDCAAS value for a mixture of products, i.e. a meal. Cited from [1].


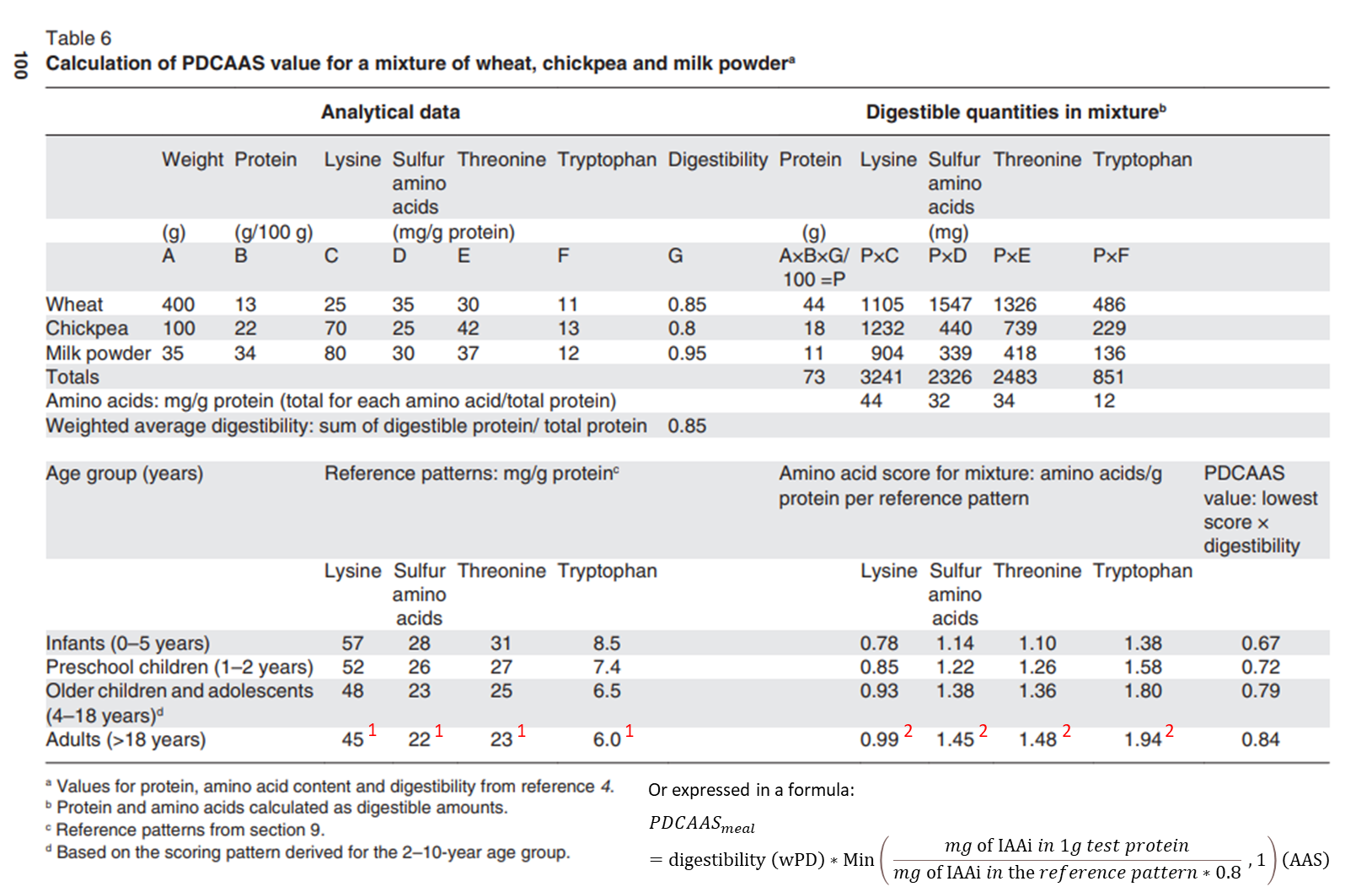


wPD: weighted protein digestibility

AAS: amino acid score

1 In the present study requirements were multiplied by 0.8, in order to compare utilizable IAAs to their requirement for 100% utilizable IAAs.

2 In the present study, the AAS was calculated as $AAS=\frac{Digestible quantity in mixture (mg/g protein)}{Requirement for adults*0.8}$.

For example for lysine this includes ${AAS}_{lys}=\frac{44}{45*0.8}=1.22$.

Note: in this example the PDCAAS value was smaller than 1. In case the PDCAAS value in the present study was larger than 1, it was truncated at 1 (see formula ${PDCAAS}_{meal}$). This truncation is needed when calculating the protein adequacy, as it corrects for the potentially limiting total amount of nitrogen [1].

Supplemental table 4. Example of the calculation of the amino acid adequacy corrected for protein digestibility and the biological value.

^
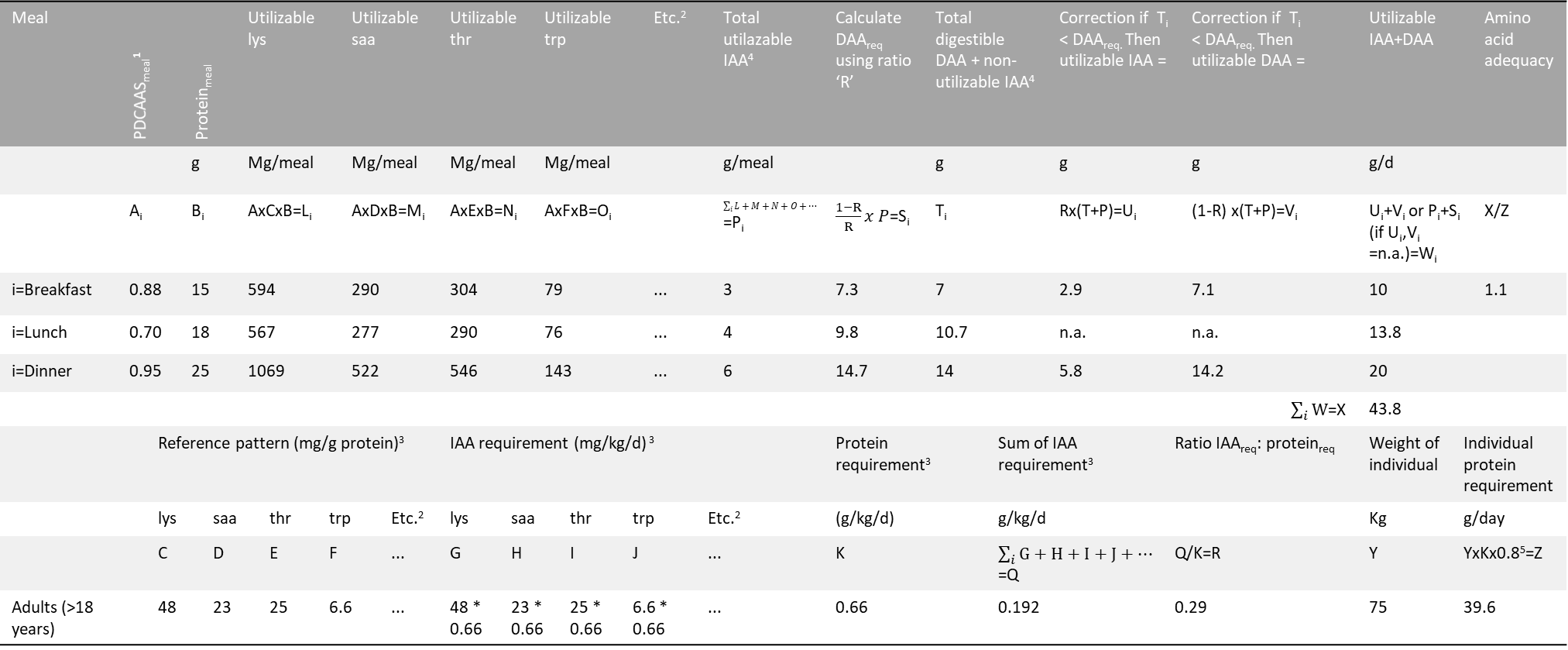
^

^1^ Calculated as in supplemental table 3, artificial data

^2^ For all essential amino acids

^3^ Reference patterns from [2]

^4^ Artificial data

^5^ As the protein digestibility of especially plant-based meals is hardly ever 100%, the protein digestibility is likely < 1. Based on literature we assume an average PD of 0.8 for the meals used in the studies of Rand et al. 2003 [3]. Once intake is corrected for the amino acid score, the protein digestibility, and the N-balance ratio of IAA:DAA, the remaining amino acids are 100% utilizable, and should be compared to the requirement for 100% utilizable protein. Therefore, when the hypothetical requirement for 100% utilizable amino acids is used, the requirement for protein intake amounts 0.66 g/kg body weight /day * 0.8 = 0.53 g/kg body weight/day.

IAA: indispensable amino acids

DAA: dispensable amino acids providing α-amino nitrogen

Req: requirement

## References

1. World Health Organization. (2007). Protein and amino acid requirements in human nutrition. *World health organization technical report series*(935): 1.
2. Food and Agriculture Organization of the United Nations., *Dietary protein quality evaluation in human nutrition: Report of an FAO Expert Consultation*, in *FAO Food and Nutrition Paper 92*. 2011: Auckland, New Zealand. p. 1-66.
3. Rand WM, Pellett PL, and Young VR (2003). Meta-analysis of nitrogen balance studies for estimating protein requirements in healthy adults. *The American Journal of Clinical Nutrition* **77**(1): 109-127. DOI: 10.1093/ajcn/77.1.109.

# Online Resource 4

The objective functions, the main constraints, and the constraints used to calculate the diet quality score (Dutch Healthy Diet index 2015 (DHD15)) are presented and explained below. This model is used iteratively to calculate an optimal diet for each of the subjects in the sample. From now on the evaluated diet is indicated with an index *j’*.

## Objective functions

Equation *(1)* is an objective function of the model, which minimizes the total GHGE of the optimized diet, expressed as a linear combination of GHGE of current diets.

| $min\left\{ F_{ghge}=\sum_{j} ghge_{j}{\cdot L}_{j} \right\}$ |  | (1) |
| --- | --- | --- |

Where $F_{ghge}$ is the total greenhouse gas emissions (GHGE) of the optimized diet, $ghge_{j}$ is the GHGE of diet *j*, and $L_{j}$is the share of diet *j* in the optimized diet.

Objective function *(2)* maximizes the Dutch Healthy Diet index 2015 (DHD15-index) which is selected to be the health score in this study [1].

| $max\left\{ F_{heal}=\sum_{c} S_{c} \right\}$ |  | (2) |
| --- | --- | --- |

Where $F_{heal}$is the health quality index of the optimized diet, and $S_{c}$ is the partial health index score of food component *c*.

## Main constraints

Constraints *(3)* are used to calculate the absolute deviation between food group consumption of the optimized and the current diet.

| $\sum_{j} q_{g,j}\cdot L_{j}{- D}_{g}^{+}+D_{g}^{-}=q_{g,j'}$ | $\forall g$ | (3) |
| --- | --- | --- |

Where $q_{g,j}$ is the consumption of food group *g* in diet *j*, $q_{g,j'}$ is the consumption level of food group *g* in the evaluated diet *j’*, $D_{g}^{+}$ is the positive deviation between the food group consumption of the optimized diet (i.e. $\sum_{j} q_{g,j}\cdot L_{j}$) and the current diet ($q_{g,j'}$), $D_{g}^{-}$ is the negative deviation between the food group consumption of the optimized diet and the current diet.

Equations *(4)* impose that for each food group the sum of the deviations is smaller than 33% of the mean current consumption in consumers, ensuring that the optimized diet remains within realistic ranges.

| $D_{g}^{+}+D_{g}^{-}\leq0.33* {mean}_{g}$ | $\forall g$ | (4) |
| --- | --- | --- |

Where ${mean}_{g}$ is the average current consumption of food group *g* in consumers (i.e. consumption>0).

Equation *(5)* is the add up constraint imposing that the sum of the shares of current diets *j* in the optimized diet does not exceed 1.

| $\sum_{j} L_{j}=1$ |  | (5) |
| --- | --- | --- |

Constraints *(6)* impose that the partial health index scores of each food component in the optimized diet are larger or equal to the partial health index scores of the same food component in the current diet, i.e. ensuring the diet will be at least as healthy as the current diet in each food component.

| $S_{c}\geq s_{c}$ | $\forall c$ | (6) |
| --- | --- | --- |

Where $S_{c}$ is the food component score that corresponds to the optimized food component intake, $s_{c}$ is the food component score that corresponds to the current food component intake.

## Modelling the partial health scores

The DHD15 index exists of 15 food components. There are four types of calculating the score for a food component in the DHD15 index, which are explained below.

The score of the components of the DHD15-index like the one presented in Supplemental figure 1 are modelled using constraints *(7)* and *(8)*. The components vegetables, fruit, whole grain products, legumes, nuts, fish, and tea are of this type. Constraint *(7)* imposes that the score increases from the minimum possible score (0) with a slope of *φ*. Constraint *(8)* imposes that the score of the component *c* is restricted to the maximum score of the specific component.

*b*

*maxS*

*Intake*

*Score (S)*

*a*

*φ*

Supplemental figure 1. Scoring function for food components of the Dutch Healthy Diet index 2015 that have a minimum intake level.

| $S_{c}\leq\varphi_{c} *intake_{c}$ |  | (7) |
| --- | --- | --- |
| $S_{c}\leq maxS_{c}$ |  | (8) |

Where *a_c_* is the maximum food component intake level with the minimum individual health score, *b_c_* is the smallest intake level that receive an individual health score of 10, *φ*_c_ is the rate of change of the score between food component intake levels *a_c_* and *b_c_*, $maxS_{c}$ is the maximum possible individual score of the component (i.e. either 5 or 10), $intake_{c}$ is the intake in gram of the component *c* in the optimized diet.

The score of the components of the DHD15 index like the one presented in Supplemental figure 2 are modelled using constraints *(9)*-*(12)*. The components red meat, processed meat, sweetened beverages and fruit juices, alcohol, and sodium are of this type.

*b*

*maxS*

*Intake*

*Score (S)*

*a*

*φ*

*b*

*maxS*

*Intake*

*Score (S)*

*a*

*φ*

Supplemental figure 2. Scoring function for food components of the Dutch Healthy Diet index 2015 that have a maximum intake level. The maximum intake level can be either 0 (left) or some positive amount a (right).

| $Intake_{c}-b_{c}\leq bigM\cdot B_{c}$ |  | (9) |
| --- | --- | --- |
| $S_{c}\leq maxS_{c}- \varphi_{c}\left( intake_{c}-a_{c} \right)+ bigN\cdot B_{c}$ |  | (10) |
| $b_{c}-intake_{c}\leq b_{c}\cdot\left( 1-B_{c} \right)$ |  | (11) |
| $S_{c}\leq maxS_{c}\cdot\left( 1-B_{c} \right)$ |  | (12) |

Where *a_c_* is the maximum food component intake level with the maximum individual health score, *b_c_* is the smallest intake level that receive the minimum individual health score, *B_c_* is a binary variable that takes the value of 1 if the food component intake becomes larger than *b_c_* (and 0 otherwise), and *bigM* and *bigN* are very large numbers.

If the binary variable *B_c_* becomes 1 then constraints *(9)* and *(10)* become not binding while constraints *(11)* and *(12)* become binding and impose that the intake is greater than *b_c_* and the individual health score is set to 0. On the contrary if *B_c_* becomes 0 then constraints *(11)* and *(12)* become not binding. Constraint *(9)* imposes that the food component intake is lower than *b_c_* and constraint *(10)* imposes that the score decreases from the maximum possible individual score with a slope *φ_c_*. For food component levels lower than a the right hand side of constraint *(10)* become more than the maximum possible individual food component score. However because of constraint *(12)* the score value is restricted to the maximum possible score of the specific component.

The score of the components of the DHD15 index like the one presented in Supplemental figure 3 are modelled using constraints *(13)*-*(17)*. The component dairy is of this type.

*maxSc*

*Score (S)*

*Intake*

*θ*

*a*

*b*

$$\text{φ}$$

*c*

*d*

Supplemental figure 3. Scoring function for food components of the Dutch Healthy Diet index 2015 that have an optimum intake level.

| $Intake_{c}-d_{c}\leq bigM\cdot B_{c}$ |  | (13) |
| --- | --- | --- |
| $S_{c}\leq maxS_{c}- \theta_{c}\left( intake-c_{c} \right)+ bigN\cdot B_{c}$ |  | (14) |
| $d_{c}-intake\leq d_{c}\cdot\left( 1-B_{c} \right)$ |  | (15) |
| $S_{c}\leq maxS_{c}\cdot\left( 1-B_{c} \right)$ |  | (16) |
| $S_{c}\leq\varphi_{c} *intake_{c}$ |  | (17) |

Where *c_c_* is the maximum food component intake level with the maximum individual health score, and *d_c_* is the minimum food component intake level with the minimum individual health score.

If the binary variable *B_c_* becomes 1 then constraints *(13)* and *(14)* become not binding while constraints *(15)* and *(16)* become binding and impose that the intake is greater than *d_c_* and the individual health score is set to 0. On the contrary if *B_c_* becomes 0 then constraints *(13)* and *(14)* become not binding. Constraint *(13)* imposes that the food component intake is lower than *d_c_* and constraint *(14)* imposes that the score decreases from the maximum possible individual score with a slope $\theta$. For food component levels lower than *c_c_* the right hand side of constraint *(14)* become more than the maximum possible individual food component score. However because of constraint *(16)* the score value is restricted to the maximum possible score of the specific component *c*. Constraint *(17)* imposes that the score increases from the minimum possible score (0) with a slope of $\varphi_{c}$, again restricted by constraint *(16)* to the maximum possible score of the specific component.

The DHD15 score comprises of ratio components like the ‘Replace refined with wholegrain products’ and ‘Replace butter and hard fats with margarines and oils’ component. The score of such components are presented in Supplemental figure 4 and are modelled using constraints *(18)*-*(20)*. To approximate ratio components in a mixed integer linear programming model we assumed that the individual score function of a ratio component remain the same between specific intake levels *(m)*. By increasing the number of intake levels we achieved a rather accurate approximation of the score function of such components.

*Score (S)*

$$e_{3}$$

*XR/YR*

$$e_{1}$$

$$e_{m}$$

${Score}_{c,4}$

*maxS*

Supplemental figure 4. Scoring function for food components of the Dutch Healthy Diet index 2015 that include a ratio.

| $e_{c,m}*YR_{c}-XR_{c}\leq bigM *(1-Y_{c,m})$ | $\forall m$ | (18) |
| --- | --- | --- |
| $\sum_{m} Y_{c,m}=1$ |  | (19) |
| $S_{c}=\sum_{m} {Score}_{c,m} *Y_{c,m}$ |  | (20) |

Where XR is the intake of the nutrient or food group that is on the numerator of the ratio component (e.g. the intake of whole grains in the optimized diet), YR is the intake of the nutrient or food group that is on the denominator (e.g. the intake of refined grains in the optimized diet), $e_{m}$ is the level of XR/YR that receives the *score_c,m_*, $Y_{c,m}$ is a binary variable that takes the value of 1 if the value of the ratio component XR/YR is larger than $e_{m}$ (and 0 otherwise).

Equation *(18)* imposes that the binary variable $Y_{c,m}$ becomes 1 if $e_{m}$ is smaller than XR/YR. Equation *(19)* ensures that only one score level can be selected. Equation *(20)* calculates the score of ratio components of the DHD15 index.

Finally, constraints *(21)* are the domain specific constraints of the decision variables.

| $L_{j}\geq0 \forall j, D_{g}^{+}\geq0 \forall g, D_{g}^{-}\geq0 \forall g, S_{c}\geq0 \forall c , B_{c} \in\left( 0,1 \right) \forall c, Y_{c,m}\in\left( 0,1 \right) \forall c,m$ |  | (21) |
| --- | --- | --- |

## References

1. Looman M, Feskens EJ, de Rijk M, Meijboom S, et al. (2017). Development and evaluation of the Dutch Healthy Diet index 2015. *Public Health Nutrition* **20**(13): 2289-2299. DOI: 10.1017/S136898001700091X.

# Online Resource 5

Supplemental table 5. Components and scoring criteria of the Dutch Healthy Diet index 2015 (DHD15-index), cited from Looman et al. 2017 [12].

| ***DHD15-index*** | **Maximum score^a^**  **(10 points)** | **Minimum score^a^**  **(0 points)** |
| --- | --- | --- |
| 1. Vegetables (g) | ≥200 | 0 |
| 2. Fruit (g) | ≥200 | 0 |
| 3a. Wholegrain products (g)  3b. Replace refined with wholegrain products | ≥90 (5 points)  No consumption of refined products or ratio wholegrain/refined ≥11 (5 points) | 0  No consumption of wholegrain products or ratio wholegrain/refined ≤0.7 |
| 4. Legumes (g) | ≥10 | 0 |
| 5. Nuts (g) | ≥15 | 0 |
| 6. Dairy products^b^ (g) | 300-450 | 0 or ≥750 |
| 7. Fish^c^ (g) | ≥15 | 0 |
| 8. Tea (g) | ≥450 | 0 |
| 9. Replace butter and hard fats with margarines and oils | No consumption of fats or ratio oils/fats ≥13 | No consumption of oils or ratio ≤0.6 |
| 10. Replace unfiltered coffee with filtered coffee | Consumption of only filtered coffee or no coffee consumption | Any consumption of unfiltered coffee |
| 11. Red meat (g) | <45 | ≥100 |
| 12. Processed meat (g) | 0 | ≥50 |
| 13. Sweetened beverages and fruit juices (g) | 0 | ≥250 |
| 14. Alcohol (g) | ≤10 | Men: ≥30  Women: ≥20 |
| 15. Sodium (g) | <1.9 | ≥3.8 |

Abbreviations: g - grams

^a^ A score above the recommended intake is 10 points, whereas an intake below is given a proportional score between 0 and 10 points.

^b^ A maximum of 40 grams cheese per day could be included.

^c^ A maximum of 4 grams lean fish per day could be included.

# Online Resource 6

Supplemental table 6. Mean (standard deviation) current total meat consumption by subgroups of age, gender, and meat quartiles, derived from the Dutch National Food Consumption Survey 2012-2016.

| Age group | Meat quartile | Meat consumption (mean (SD)) (g/day) | | | |
| --- | --- | --- | --- | --- | --- |
|  |  | Men | N | Women | N |
| 18-50 years | 1 | 9.3 (11.2) | 214 | 7.6 (10.7) | 306 |
|  | 2 | 54.3 (12.2) | 278 | 55.0 (11.8) | 256 |
|  | 3 | 97.8 (13.5) | 271 | 98.2 (13.6) | 254 |
|  | 4 | 186.0 (60.4) | 333 | 188.3 (56.2) | 310 |
| 50-79 years | 1 | 10.4 (11.3) | 186 | 8.7 (11.3) | 249 |
|  | 2 | 55.7 (12.6) | 222 | 54.7 (12.5) | 204 |
|  | 3 | 98.5 (13.8) | 276 | 98.4 (13.8) | 250 |
|  | 4 | 176.4 (52.7) | 364 | 185.9 (64) | 327 |

# Online Resource 7

Supplemental figure 5. Average consumed digestible indispensable amino acid (IAA) (mg/g protein) per meal, and the requirement (horizontal bars) of the corresponding IAA for Dutch **men (18-50 y)**. The dotted horizontal bars show the original requirement, as obtained from Table 5 of [1]. The solid horizontal bars show the requirement corrected for digestibility (multiplied by 0.8). Note that the IAA numbers provide information on protein quality, i.e. the figure shows which IAA is lower than its requirement and may therefore be more often the limiting amino acid when defining the PDCAAS. However, this figure does not provide information on protein adequacy, as adequacy is a combination of protein quality and protein quantity. Moreover, these numbers are not yet corrected for the required ratio of IAAs:DAAs in each meal. Therefore, if the DAAs are limiting, IAAs available for utilization are reduced.

Supplemental figure 6. Average consumed digestible indispensable amino acid (IAA) (mg/g protein) per meal, and the requirement (horizontal bars) of the corresponding IAA for Dutch **women (18-50 y)**. The dotted horizontal bars show the original requirement, as obtained from Table 5 of [1]. The solid horizontal bars show the requirement corrected for digestibility (multiplied by 0.8). Note that the IAA numbers provide information on protein quality, i.e. the figure shows which IAA is lower than its requirement and may therefore be more often the limiting amino acid when defining the PDCAAS. However, this figure does not provide information on protein adequacy, as adequacy is a combination of protein quality and protein quantity. Moreover, these numbers are not yet corrected for the required ratio of IAAs:DAAs in each meal. Therefore, if the DAAs are limiting, IAAs available for utilization are reduced.

Supplemental figure 7. Average consumed digestible indispensable amino acid (IAA) (mg/g protein) per meal, and the requirement (horizontal bars) of the corresponding IAA for Dutch **men (51-79 y)**. The dotted horizontal bars show the original requirement, as obtained from Table 5 of [1]. The solid horizontal bars show the requirement corrected for digestibility (multiplied by 0.8). Note that the IAA numbers provide information on protein quality, i.e. the figure shows which IAA is lower than its requirement and may therefore be more often the limiting amino acid when defining the PDCAAS. However, this figure does not provide information on protein adequacy, as adequacy is a combination of protein quality and protein quantity. Moreover, these numbers are not yet corrected for the required ratio of IAAs:DAAs in each meal. Therefore, if the DAAs are limiting, IAAs available for utilization are reduced.

Supplemental figure 8. Average consumed digestible indispensable amino acid (IAA) (mg/g protein) per meal, and the requirement (horizontal bars) of the corresponding IAA for Dutch **women (51-79 y)**. The dotted horizontal bars show the original requirement, as obtained from Table 5 of [1]. The solid horizontal bars show the requirement corrected for digestibility (multiplied by 0.8). Note that the IAA numbers provide information on protein quality, i.e. the figure shows which IAA is lower than its requirement and may therefore be more often the limiting amino acid when defining the PDCAAS. However, this figure does not provide information on protein adequacy, as adequacy is a combination of protein quality and protein quantity. Moreover, these numbers are not yet corrected for the required ratio of IAAs:DAAs in each meal. Therefore, if the DAAs are limiting, IAAs available for utilization are reduced.

## References

1. Food and Agriculture Organization of the United Nations., *Dietary protein quality evaluation in human nutrition: Report of an FAO Expert Consultation*, in *FAO Food and Nutrition Paper 92*. 2011: Auckland, New Zealand. p. 1-66.

# Online Resource 8


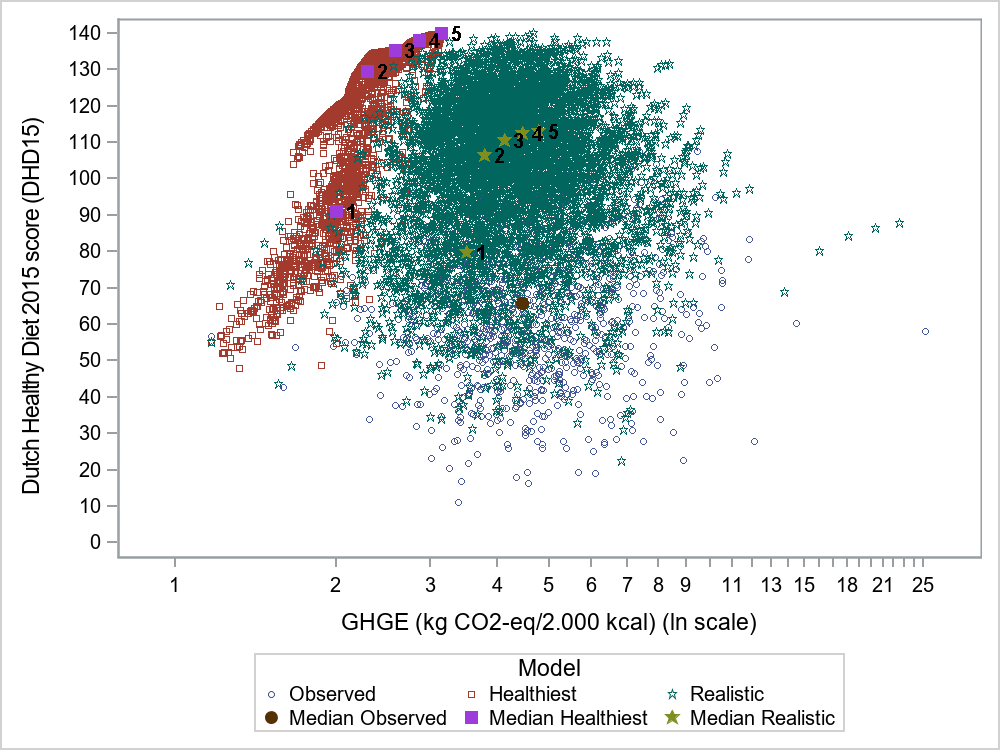


Supplemental figure 9. Trade-off between maximizing dietary health (DHD15) and minimizing GHGE among Dutch **women (18-50 y)**. Filled symbols represent the medians for all 10 models (‘healthiest’ (phase 2) and ‘realistic’ (phase 3), with 5 runs each) and for the current diet. Open symbols represent individual data of the current and modelled diets. ‘Healthiest diets’ have no restriction on deviation to the current diet. In ‘realistic diets’ deviation is restricted to 33% of current consumption per food group.


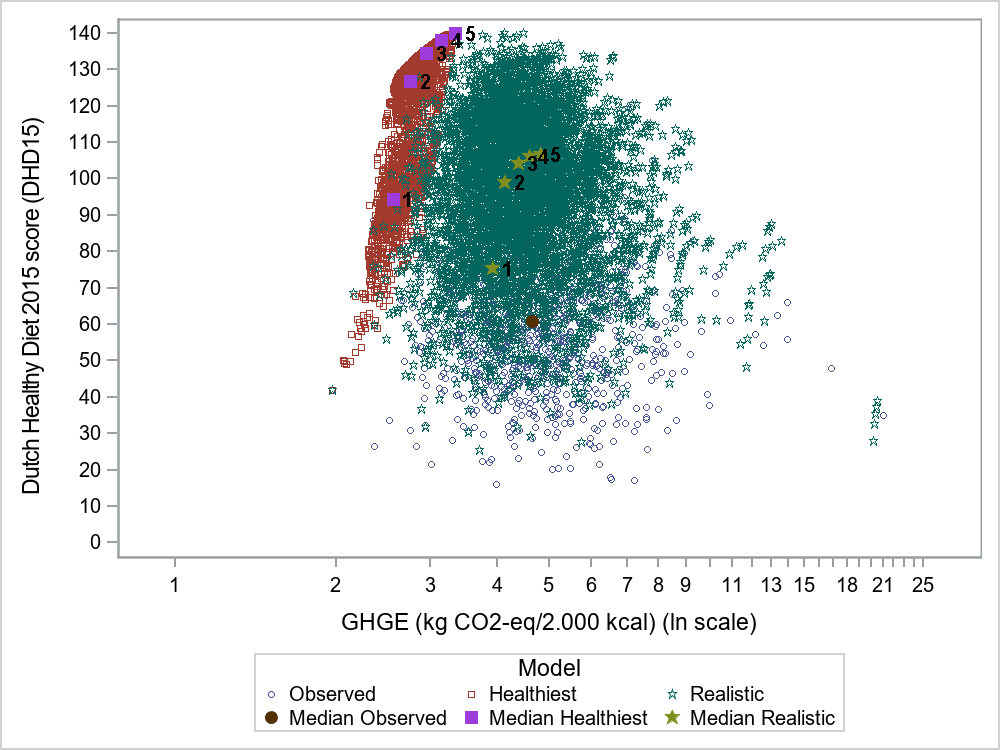


Supplemental figure 10. Trade-off between maximizing dietary health (DHD15) and minimizing GHGE among Dutch **men (50-79 y)**. Filled symbols represent the medians for all 10 models (‘healthiest’ (phase 2) and ‘realistic’ (phase 3), with 5 runs each) and for the current diet. Open symbols represent individual data of the current and modelled diets. ‘Healthiest diets’ have no restriction on deviation to the current diet. In ‘realistic diets’ deviation is restricted to 33% of current consumption per food group.


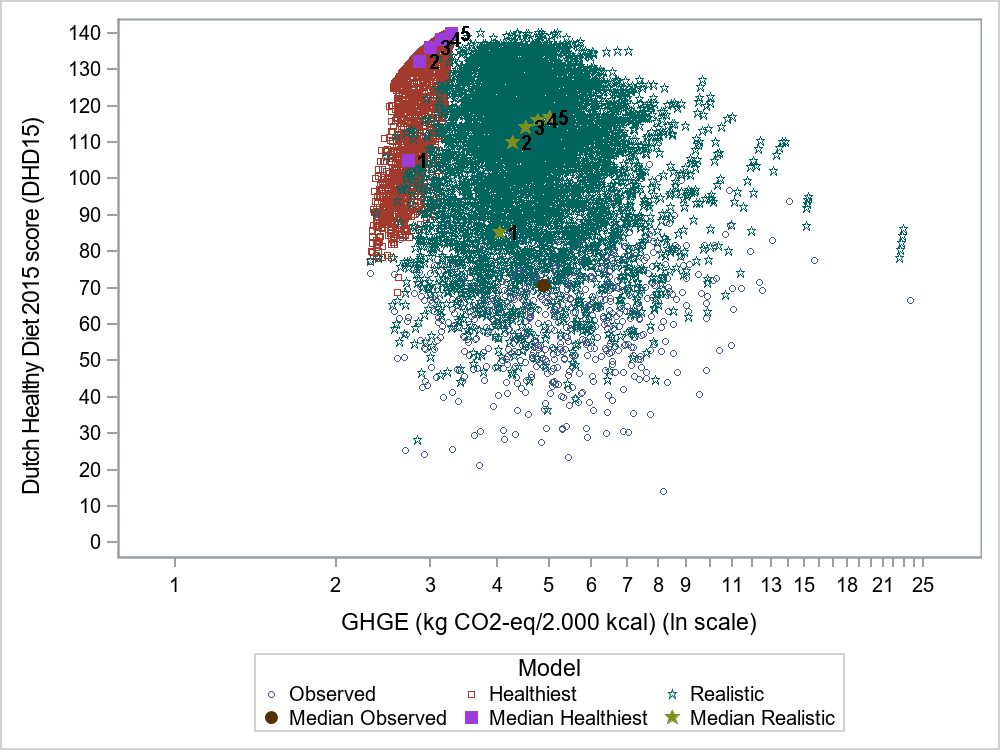


Supplemental figure 11. Trade-off between maximizing dietary health (DHD15) and minimizing GHGE among Dutch **women (50-79 y)**. Filled symbols represent the medians for all 10 models (‘healthiest’ (phase 2) and ‘realistic’ (phase 3), with 5 runs each) and for the current diet. Open symbols represent individual data of the current and modelled diets. ‘Healthiest diets’ have no restriction on deviation to the current diet. In ‘realistic diets’ deviation is restricted to 33% of current consumption per food group.

# Online Resource 9


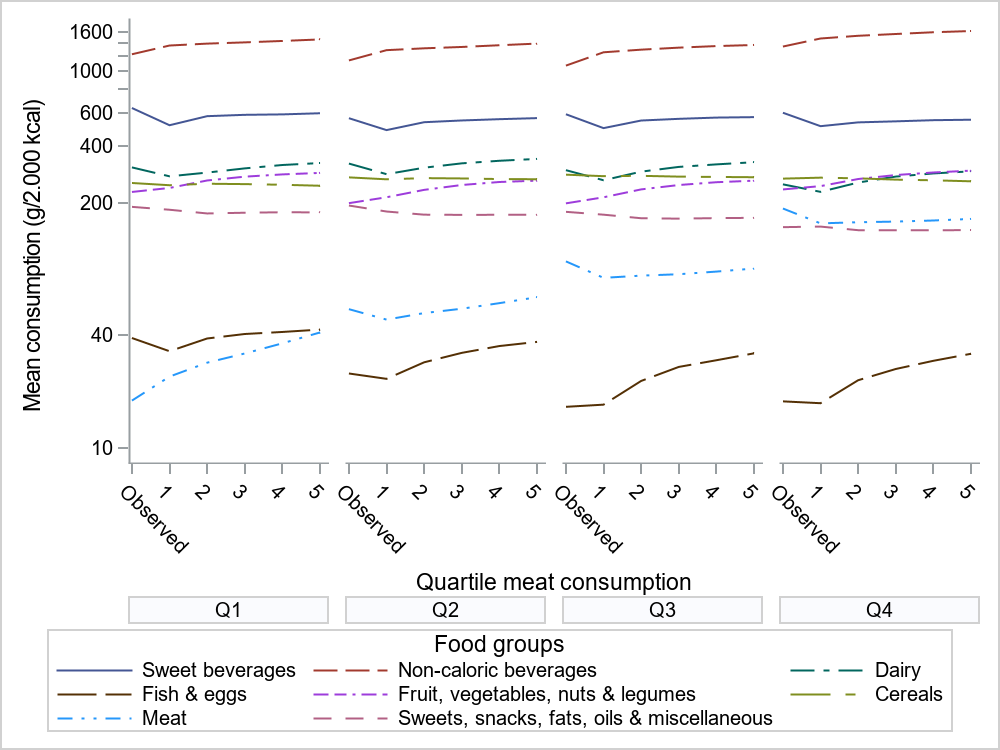


Supplemental figure 12. Mean food group consumption in g/2.000 kcal for **men (18-50 y)** by quartile of meat consumption, current diets and ‘realistic diets’, i.e. deviation restricted to 33% of current consumption per food group, per subgroup of age and gender. The lines in between are meant to recognise the pattern and they are not linear interpolations.


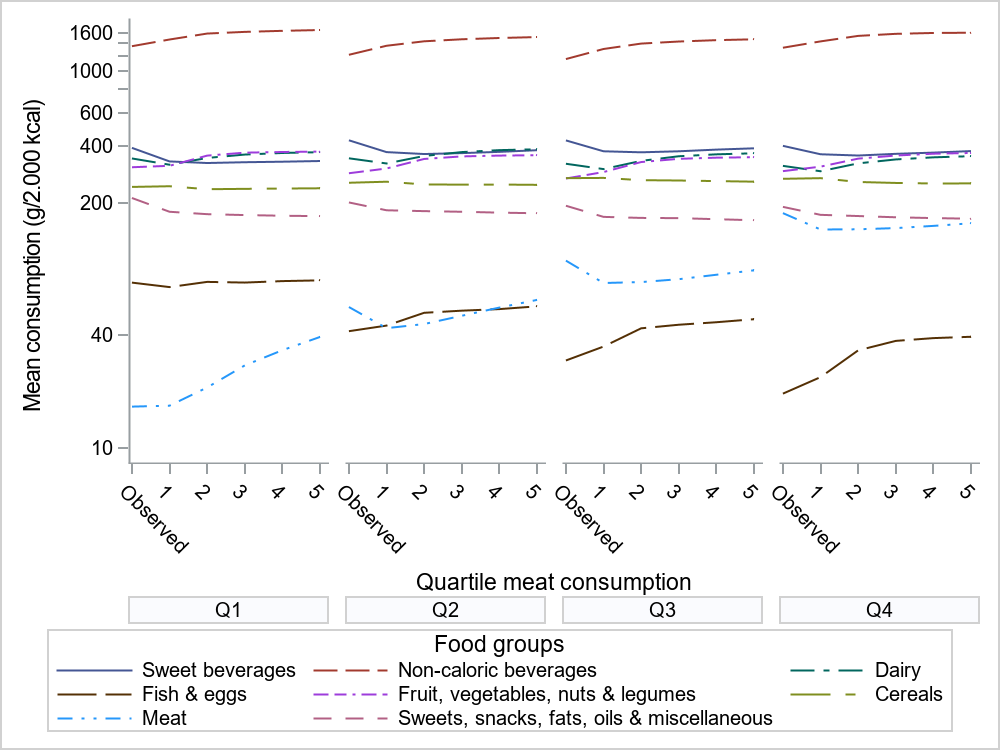


Supplemental figure 13. Mean food group consumption in g/2.000 kcal for **men (50-79 y)** by quartile of meat consumption, current diets and ‘realistic diets’, i.e. deviation restricted to 33% of current consumption per food group, per subgroup of age and gender. The lines in between are meant to recognise the pattern and they are not linear interpolations.


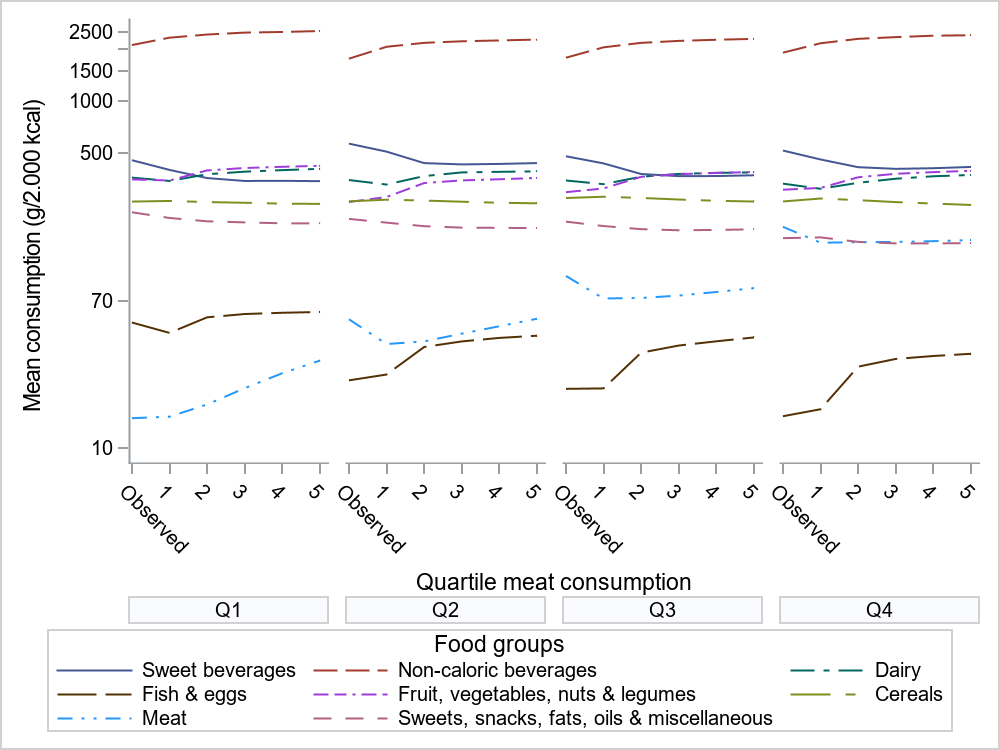


Supplemental figure 14. Mean food group consumption in g/2.000 kcal for **women (18-50 y)** by quartile of meat consumption, current diets and ‘realistic diets’, i.e. deviation restricted to 33% of current consumption per food group, per subgroup of age and gender. The lines in between are meant to recognise the pattern and they are not linear interpolations.


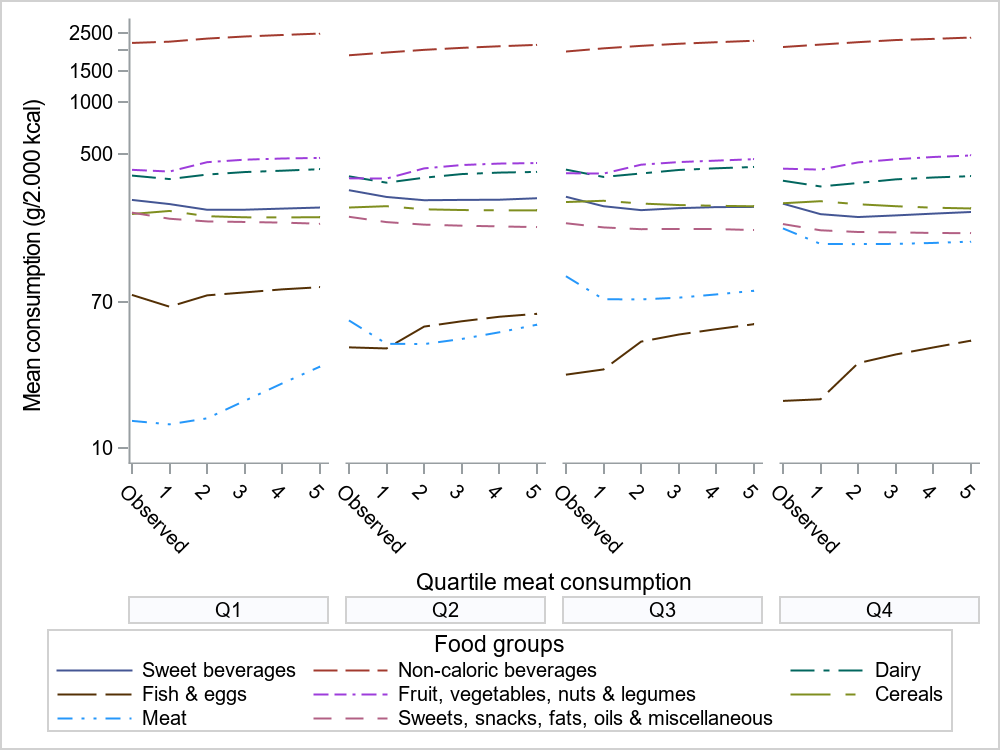


Supplemental figure 15. Mean food group consumption in g/2.000 kcal for **women (50-79 y)** by quartile of meat consumption, current diets and ‘realistic diets’, i.e. deviation restricted to 33% of current consumption per food group, per subgroup of age and gender. The lines in between are meant to recognise the pattern and they are not linear interpolations.

# Online Resource 10

Supplemental table 7. Nutrient intakes (mean) per 2.000 kcal of the current diets and of the second run of the ‘realistic diets’ (phase 3)(optimized), i.e. deviation restricted to 33% of current consumption per food group.

|  |  | Q1 (~9 g) | | Q2 (~55 g) | | Q3 (~98 g) | | Q4 (~184 g) | |
| --- | --- | --- | --- | --- | --- | --- | --- | --- | --- |
| Population subgroup | Nutrient | Current | Optimized | Current | Optimized | Current | Optimized | Current | Optimized |
| Men (18-50 y) | Energy % protein | 12.7 | 12.7 | 13.4 | 13.3 | 14.6 | 14.1 | 18.3 | 17.2 |
|  | % plant protein | 54.5 | 55.1 | 46.7 | 50.1 | 41.6 | 46.1 | 31.2 | 35.7 |
|  | Calcium (mg) | 926 | 882 | 892 | 877 | 855 | 842 | 802 | 812 |
|  | Iron (mg) | 8.6 | 9.0 | 8.7 | 9.0 | 9.1 | 9.3 | 10.3 | 10.2 |
|  | Vitamin B12 (µg) | 3.35 | 3.21 | 3.46 | 3.30 | 3.64 | 3.56 | 4.60 | 4.38 |
| Men (50-79 y) | Energy % protein | 13.7 | 14.0 | 14.0 | 14.1 | 15.1 | 14.8 | 18.3 | 17.5 |
|  | % plant protein | 47.6 | 48.7 | 42.0 | 45.8 | 38.3 | 42.8 | 30.4 | 34.5 |
|  | Calcium (mg) | 1009 | 1004 | 956 | 968 | 923 | 936 | 897 | 913 |
|  | Iron (mg) | 9.7 | 9.9 | 9.4 | 9.8 | 10.1 | 10.3 | 11.2 | 11.2 |
|  | Vitamin B12 (µg) | 4.89 | 4.70 | 4.34 | 4.39 | 4.42 | 4.66 | 5.60 | 5.93 |
| Women (18-50 y) | Energy % protein | 13.7 | 14.0 | 14.0 | 14.2 | 15.3 | 15.1 | 18.7 | 17.9 |
|  | % plant protein | 52.7 | 54.0 | 46.2 | 50.5 | 39.9 | 45.5 | 30.3 | 35.6 |
|  | Calcium (mg) | 1094 | 1096 | 1010 | 1044 | 1010 | 1033 | 950 | 966 |
|  | Iron (mg) | 9.4 | 9.7 | 9.1 | 9.5 | 9.9 | 10.1 | 10.4 | 10.5 |
|  | Vitamin B12 (µg) | 3.97 | 4.08 | 3.63 | 3.81 | 3.97 | 3.91 | 4.48 | 4.40 |
| Women (50-79 y) | Energy % protein | 14.5 | 14.7 | 14.5 | 14.6 | 16.0 | 15.6 | 19.3 | 18.3 |
|  | % plant protein | 45.5 | 47.2 | 43.2 | 46.5 | 36.7 | 41.5 | 30.0 | 34.1 |
|  | Calcium (mg) | 1182 | 1159 | 1083 | 1084 | 1108 | 1092 | 1019 | 1014 |
|  | Iron (mg) | 10.1 | 10.4 | 10.3 | 10.9 | 11.0 | 11.5 | 12.2 | 12.7 |
|  | Vitamin B12 (µg) | 5.07 | 5.08 | 3.93 | 5.13 | 4.61 | 5.96 | 5.86 | 6.99 |
